# Revision of the freshwater crabs of the *Johora tahanensis* (Bott, 1966) species group (Crustacea, Brachyura, Potamidae), with a key to the genus

**DOI:** 10.3897/zookeys.994.56810

**Published:** 2020-11-17

**Authors:** Peter K. L. Ng

**Affiliations:** 1 Lee Kong Chian Natural History Museum, National University of Singapore, 2 Conservatory Drive, Singapore 117377, Singapore National University of Singapore Singapore Singapore

**Keywords:** Freshwater crab, new species, Peninsular Malaysia, Potamoidea, taxonomy, Thailand

## Abstract

The taxonomy of the potamid freshwater crabs of the *Johora tahanensis* (Bott, 1966) species group (Potamoidea) is revised. Seven species are recognised, all from Peninsular Malaysia and southern Thailand, three of which are described as new. The three new species were previously identified as *J. tahanensis* but can be distinguished by characters of the carapace, male first gonopod, and vulva. A revised key to the 18 recognised species of *Johora* Bott, 1966, is provided.

## Introduction

Six genera of potamid freshwater crabs are known from the Malay Peninsula (Peninsular Malaysia, Thailand south of the Isthmus of Kra, and Singapore): *Baccazia* Ng, 2018a, *Demanietta* Bott, 1966, *Gempala* Ng & Ahmad, 2016, *Johora* Bott, 1966, *Stoliczia* Bott, 1966, and *Terrapotamon* Ng, 1986a (see Ng 1988, 2018a, b; Yeo et al. 1999; Ng and Ahmad 2016; Ng 2018). *Gempala* and *Baccazia* are unusual in that the terminal lobe of their mandibular palp is bilobed whereas in the other genera (and all other potamids), the terminal lobe is simple (Ng and Ahmad 2016; Ng 2018a). *Stoliczia* and *Terrapotamon* are distinct in having only a short flagellum (often absent) on the exopod of the third maxilliped (Ng 1986a, 1988, 2004; Lheknim and Ng 2016; Promdam et al. 2017). Members of *Demanietta* and *Johora* possess a simple terminal lobe on the mandibular palp and the exopod of the third maxilliped has a long flagellum. *Demanietta* is known from eastern Myanmar and Thailand, but reaches only to Phuket (Yeo et al. 1999; Ng 2018b); whereas *Johora* occurs from southernmost Thailand to Singapore (Ng 1988, 2004; Leelawathanagoon et al. 2005; Yeo et al. 2007).

*Johora* is one of the most diverse of these Malayan genera, with 15 known species: *J. aipooae* (Ng, 1986a), *J. counsilmani* (Ng, 1985), *J. gapensis* (Bott, 1966), *J. grallator* Ng, 1988, *J. gua* Yeo, 2001, *J. hoiseni* Ng & Takeda, 1992, *J. intermedia* (Ng, 1986b), *J. johorensis* (Roux, 1936), *J. murphyi* (Ng, 1986b), *J. punicea* (Ng, 1985), *J. singaporensis* (Ng, 1986b), *J. tahanensis* (Bott, 1966), *J. thaiana* Leelawathanagoon, Lheknim & Ng, 2005, *J. thoi* Ng, 1990, and *J. tiomanensis* (Ng & Tan, 1984) (Ng 1987, 1988, 1990; Ng and Takeda 1992; Yeo 2001; Leelawathanagoon et al. 2005; Ng et al. 2008).

The phylogenetic study by Yeo et al. (2007) showed that *Johora* is a monophyletic genus but three subclades were discerned. One of these subclades is the *J. tahanensis* species group (with *J. tahanensis*, *J. hoiseni* and *J. thoi*), which is distributed from central to northern Peninsular Malaysia and southern Thailand (Yeo et al. 2007: fig. 1). The present study revises the taxonomy of the *J. tahanensis* species group, and three new species are recognised. The three new species have all originally been referred to *J. tahanensis* mainly because of their relatively large adult size and general shape of the G1 structure. Detailed comparisons show they are morphologically distinct, and with discreet distributions, and *J. tahanensis* s. str. is not a wide-ranging species in Peninsular Malaysia. The *J. tahanensis* species group now contains seven species, including *J. thaiana* and the three new species described here. A revised key to all *Johora* species is also provided.

## Materials and methods

Measurements provided are of the maximum carapace width and length (in millimetres), respectively. The abbreviations G1 and G2 are used for the male first and second gonopods, respectively. The terminology used follows Ng (1988), Guinot et al. (2013) and Davie et al. (2015). The Malay words Pulau, Gunung and Sungei are used for island, mountain and river, respectively. Material examined is deposited in the Zoological Reference Collection of the Lee Kong Chian Natural History Museum, National University of Singapore (**ZRC**).

A full description is given for *Johora tahanensis*; for all other taxa, only diagnoses are provided.

## Systematics

### Family Potamidae Ortmann, 1896

#### Subfamily Potamiscinae Bott, 1970 sensu Yeo and Ng (2004)

##### Genus Johora

Taxon classificationAnimaliaDecapodaPotamidae

Bott, 1966

A9062979-ED11-52B1-8751-6DC9D8AB3C57

###### Type species.

Potamon (Potamon) johorense Roux, 1936, by original designation.

###### Comparative material.

*Johora johorensis* (Roux, 1936): 1 male, 1 female, 1 juvenile (ZRC 2019.1054), Sungei Pulai, Gunung Pulai, Pulai, clear waters, sandy, and large rock substrate; shallow to waist deep waters with low vegetation cover on sides, in forest, 1°35'31.1"N, 103°31'10.7"E, Johor, coll. BY Lee et al., 23 June 2019. *Johora intermedia* Ng, 1986b: holotype male (19.8 × 15.1 mm) (ZRC 1984. 6529), stream near Bentong, Pahang, ca. 3°27'36"N, 101°53'31"E, ca. 600 m a.s.l., coll. Tweedie MWF, 12 July 1935; paratypes 26 males, 13 females, 17 juveniles (ZRC 1984. 6530–6585), same data as holotype; 7 males, 4 females, 1 juvenile (ZRC 1989.2171–2182), Gombak Forest, Selangor, coll. Ng PKL, 9 June 1987; 2 males, 2 females, 1 juvenile (ZRC 2001.1002), stream at Engkabau Trail, river tract, Forest Research Institute of Malaysia (FRIM), forest reserve, Kepong, Selangor, coll. Leong TM et al., September 2000; 3 males (ZRC 2003.0327), Sungei Kroh and Engkabau Trail, stream in Forest Research Institute of Malaysia (FRIM), Kepong, Selangor, coll. Leong TM & Lim KKP, 22–27 November 2002; 3 males (ZRC 2001.2283), Genting Highlands, Pahang, coll. Leong TM, 24 October 2001; 1 female (ZRC 2013.1819), Genting Highlands, Pahang, coll. Barlow HS, 9 April 2013; 2 males, 2 females (ZRC 2018.0690), Genting Tea Estate, Pahang, 3°21'24.9"N, 101°47'42.2"E, 670 m, coll. Barlow HS, 2000s; 4 males (ZRC 2002.242), in roadside seepage, on road to Jeriau Falls, Fraser’s Hill, Pahang, coll. Leong TM, 24 February 2002; 2 males (ZRC 2016.003), under rocks, first stream on road towards Raub, descending from Fraser’s Hill, Pahang, coll. Lai JCY, 6 December 2015; 2 males (ZRC 2020.0073), shaded stream along road, Fraser’s Hill, Jalan Telekom, Pahang, 3.715485, 101.747604, coll. Lai JCY, December 2015; 1 male, 1 juvenile female (ZRC 2016.0002), Jeriau Falls, Fraser’s Hill, Pahang, coll. Lai JCY & Hogg AH, 5 December 2015; 1 male (ZRC 2020.0354), Jeriau Falls, Fraser’s Hill, Pahang, coll. Hogg AH, 30 May 2016. *Johora gapensis* (Bott, 1966): 2 males, 2 females (ZRC 1995.349), in shallow leaf litter stream, The Gap, Fraser’s Hill, coll. Lim KKP, 9 June 1990; 1 male (ZRC 2002.0587), Gunung Bunga Buah, Genting Highlands, 3°22'30.0"N, 101°44'23.7"E, Pahang, coll. Lim KKP, 4 July 2002; 1 male (ZRC 2020.0074), along path to main waterfall, Jerijau Waterfall, Fraser’s Hill, Pahang, 3.724534, 101.714471, coll. Lai JCY, December 2015. All locations in Peninsular Malaysia. For material of other *Johora* species, see Ng (1985, 1987, 1990), Ng and Takeda (1992), Yeo et al. (1999), Yeo (2001) and Leelawathanagoon et al. (2005).

###### Remarks.

Members of the *J. tahanensis* species group are distinct from the nominate species group (*J. johorensis*, *J. gapensis*, *J. tiomanensis*, *J. counsilmani*, *J. intermedia*, *J. murphyi* and *J. singaporensis*) (sensu Yeo et al. 2007) and can be distinguished by the following suite of characters: the epibranchial tooth is prominent, clearly demarcated from the external orbital tooth by a distinct cleft (Fig. 2B–H); the frontal margin is relatively wide (Fig. 2B–H); the epigastric cristae are prominently anterior of the postorbital cristae (Fig. 2B–H); the postorbital crista is sharp and prominent, with the lateral part clearly reaching at least the beginning of the cervical groove (Fig. 2B–H); the lateral margins of the posterior margin of the epistome are obliquely sloping (Fig. 3B–H); the third maxilliped is covered with dense, long setae, and the merus and ischium are proportionately longer (Figs 3B–H, 4B); the male pleon is proportionately more elongate with somite 3 relatively less wide (Figs 5B–H, 6D, 7D); the G1 terminal segment is long to very long and often covered with numerous setae (e.g., Fig. 8A); and the adult female pleon is ovate (Fig. 13B–H). For members of the *J. johorensis* species group, the epibranchial tooth is usually lower and demarcated from the external orbital tooth by a shallow or narrow cleft (Fig. 2A); the frontal margin is relatively less wide (Fig. 2A); the epigastric cristae are only slightly anterior of the postorbital cristae (Fig. 2A); the postorbital crista is uneven and not sharp and do not clearly extend to the cervical groove (Fig. 2A); the lateral margins of the posterior margin of the epistome are horizontal and subparallel with the frontal margin (Fig. 3A); the setae on the third maxilliped are shorter and usually less dense, and in the smaller taxa, the merus and ischium are proportionately shorter (Figs 3A, 4A); the male pleon is broadly triangular with somite 3 relatively wider (Fig. 5A); the G1 terminal segment is short to long (e.g., Ng 1987: figs 2F, I, 3A, 6D); and the adult female pleon is almost round (Fig. 13A).

In recent years, the structure of the vulva has proven useful in potamid taxonomy for some groups, but for *Johora* species, its morphology appears to be relatively conservative. The one exception is *J. thoi*, where the lateral sternal vulvar cover projects obliquely anteriorly as a large triangular plate (Fig. 14C), quite different from the simpler structures of congeners (Fig. 14A, B, D–H).

**Figure 1.**
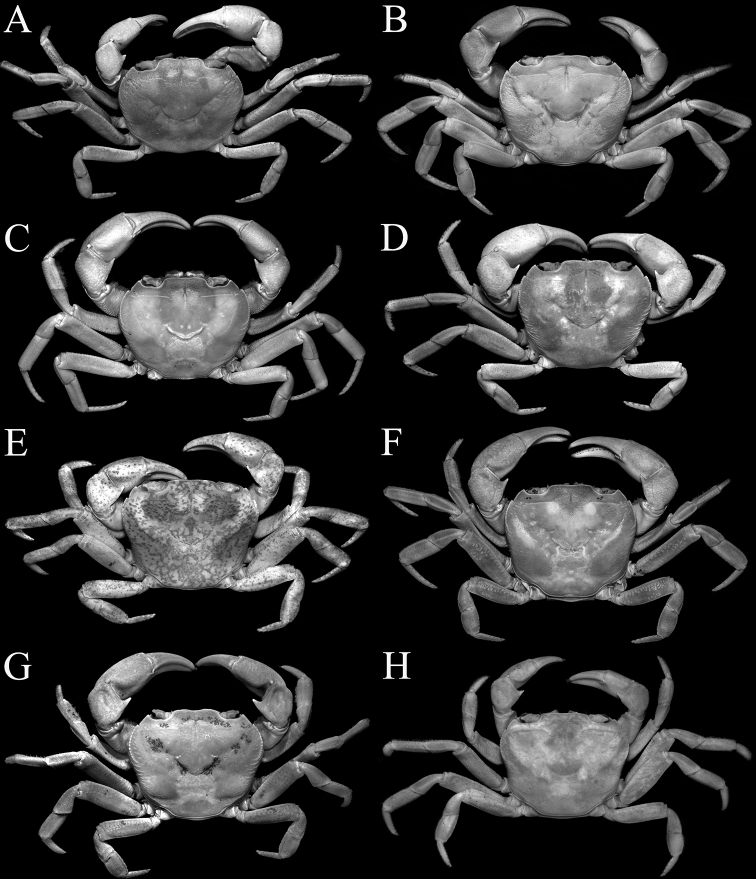
Overall dorsal view **A***Johora intermedia* Ng, 1986, holotype male (19.8 × 15.1 mm) (ZRC 1984.6529), Pahang **B***J. tahanensis* (Bott, 1966), male (30.7 × 25.5 mm) (ZRC 1984.6795), Pahang **C***J. thoi* Ng, 1990, holotype male (41.2 × 33.3 mm) (ZRC 1989.2249), Terengganu **D***J. hoiseni* Ng & Takeda, 1992, holotype male (25.1 × 21.1 mm) (ZRC 1984.6673), Kelantan **E***J. thaiana* Leelawathanagoon, Lheknim & Ng, 2005, paratype male (22.2 × 18.6 mm) (ZRC 2006.0052), Thailand **F***J. booliati* sp. nov., holotype male (34.6 × 30.0 mm) (ZRC 2020.0072), Pahang **G***J. erici* sp. nov., holotype male (38.3 × 32.0 mm) (ZRC 2020.0360), Perak **H***J. michaeli* sp. nov., holotype male (22.7 × 19.2 mm) (ZRC 2010.0047), Terengganu.

The molecular study by Yeo et al. (2007) tested three members of the *J. tahanensis* species group: “*J. tahanensis*” from Perak (ZRC 1995.268), *J. hoiseni* from Kelantan (ZRC1984.6674–6755, ZRC 1984.7683–7687), and *J. thoi* from Terengganu (ZRC 2001.1167), with the taxa coming out in one clade. The “*J. tahanensis*” from Perak (ZRC 1995.268) is here reidentified as a new species, *J. erici* sp. nov.

The last key to the genus was constructed in 1988 and in view of the additional species described since, there is a necessity to update this, and a revised key is presented here.

**Figure 2.**
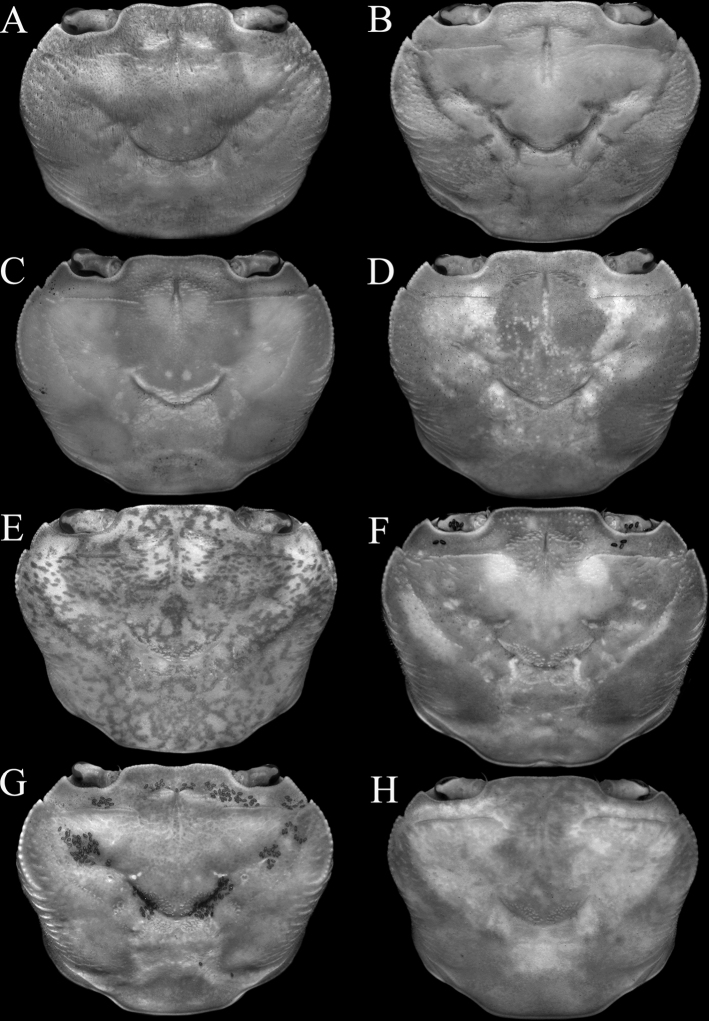
Dorsal view of carapace **A***Johora intermedia* Ng, 1986, holotype male (19.8 × 15.1 mm) (ZRC 1984.6529), Pahang **B***J. tahanensis* (Bott, 1966), male (30.7 × 25.5 mm) (ZRC 1984.6795), Pahang **C***J. thoi* Ng, 1990, holotype male (41.2 × 33.3 mm) (ZRC 1989.2249) Terengganu **D***J. hoiseni* Ng & Takeda, 1992, holotype male (25.1 × 21.1 mm) (ZRC 1984.6673), Kelantan **E***J. thaiana* Leelawathanagoon, Lheknim & Ng, 2005, paratype male (22.2 × 18.6 mm) (ZRC 2006.0052), Thailand **F***J. booliati* sp. nov., holotype male (34.6 × 30.0 mm) (ZRC 2020.0072), Pahang **G***J. erici* sp. nov., holotype male (38.3 × 32.0 mm) (ZRC 2020.0360), Perak **H***J. michaeli* sp. nov., holotype male (22.7 × 19.2 mm) (ZRC 2010.0047), Terengganu.

##### Johora tahanensis

Taxon classificationAnimaliaDecapodaPotamidae

Bott, 1966

CEDC1AC6-17E4-5B1D-9851-FF5A4A16DA00

Figures 1B , 2B , 3B , 4B , 5B , 8A–D , 12B , 13B , 14B

Potamiscus (Johora) johorensis tahanensis Bott, 1966: 495 (part), pl. 21 fig. 15.
Stoliczia (Johora) johorensis tahanensis – Bott 1970: 181 (part), pl. 50 fig. 50.
Johora tahanensis – Ng 1987: 33 (part), fig. 9A; Ng 1988: 42 (part), fig. C, F; Ng and Takeda 1992: 107 (part); Ng 2004: 321; Ng and Yeo 2007: 102; Ng et al. 2008: 163; Cumberlidge et al. 2009: table.
Stoliczia johorensis tahanensis – Takeda 1987: 92, pl. X (center).

###### Material examined.

13 males (largest 27.2 × 22.9 mm), 13 females (largest 24.4 × 21.0 mm), 4 juveniles (ZRC 1984.6764–6793), Kuala Tahan, Taman Negara National Park, Pahang, coll. Tweedie MWF, April 1940; 1 male (30.7 × 25.5 mm), 2 females (ZRC 1984.6795–6797), rivulet of Sungei Tahan, Kuala Tahan, Taman Negara National Park, Pahang, coll. Alfred ER, 23 March 1956; 1 male (ZRC 1989.2086), Changah Siveh, Sungei Tahan, Taman Negara National Park, Pahang, coll. Alfred ER, 3 March 1948; 1 female (ZRC 1989.3749), Jenka, Sungei Tekam, Pahang, 4°13'N, 102°39'E, coll. Lim RP, 19 March 1981; 1 young male, 1 female (ZRC 1989.2144–2145), from University of Malaya collections, 1960s; 2 juveniles (ZRC 1989.3358–3359), station F63/20, tributary of Sungei Telom (= Sungei Telum), Pahang, coll. University of Malaya, 9 March 1963; 1 female (ZRC 1989.3686), coll. University of Malaya, no date. All locations in Peninsular Malaysia.

###### Diagnosis.

**Figure 3.**
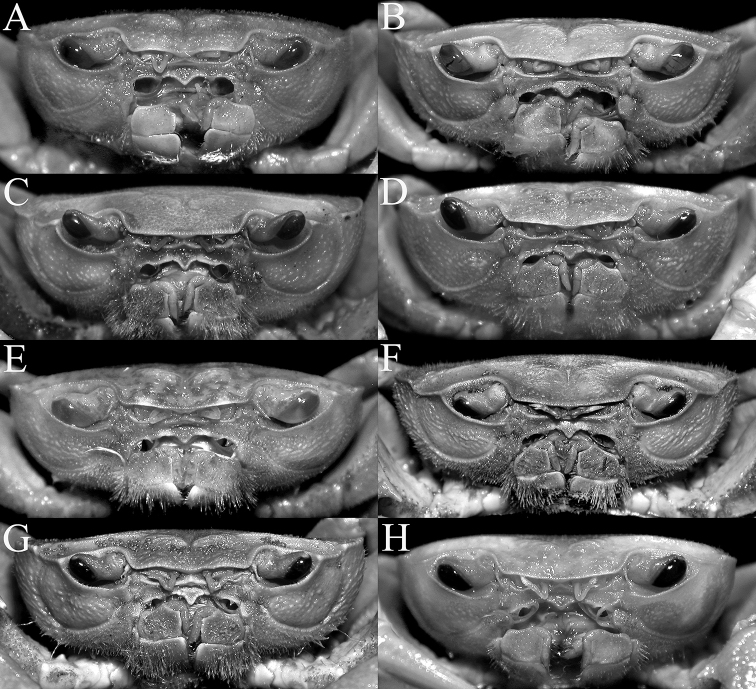
Frontal view of cephalothorax **A***Johora intermedia* Ng, 1986, holotype male (19.8 × 15.1 mm) (ZRC 1984.6529), Pahang **B***J. tahanensis* (Bott, 1966), male (30.7 × 25.5 mm) (ZRC 1984.6795), Pahang **C***J. thoi* Ng, 1990, holotype male (41.2 × 33.3 mm) (ZRC 1989.2249), Terengganu **D***J. hoiseni* Ng & Takeda, 1992, holotype male (25.1 × 21.1 mm) (ZRC 1984.6673), Kelantan **E***J. thaiana* Leelawathanagoon, Lheknim & Ng, 2005, paratype male (22.2 × 18.6 mm) (ZRC 2006.0052), Thailand **F***J. booliati* sp. nov., holotype male (34.6 × 30.0 mm) (ZRC 2020.0072), Pahang **G***J. erici* sp. nov., holotype male (38.3 × 32.0 mm) (ZRC 2020.0360), Perak **H***J. michaeli* sp. nov., holotype male (22.7 × 19.2 mm) (ZRC 2010.0047), Terengganu.

**Figure 4.**
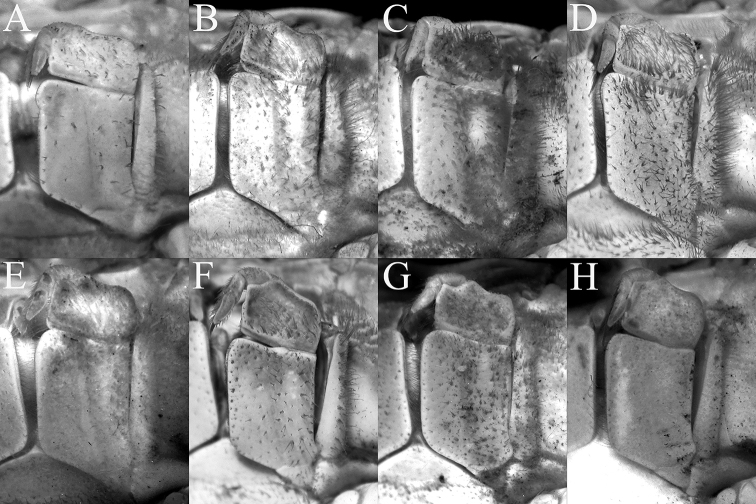
Left third maxilliped **A***Johora intermedia* Ng, 1986, holotype male (19.8 × 15.1 mm) (ZRC 1984.6529), Pahang **B***J. tahanensis* (Bott, 1966), male (30.7 × 25.5 mm) (ZRC 1984.6795), Pahang **C***J. thoi* Ng, 1990, holotype male (41.2 × 33.3 mm) (ZRC 1989.2249), Terengganu **D***J. hoiseni* Ng & Takeda, 1992, holotype male (25.1 × 21.1 mm) (ZRC 1984.6673), Kelantan **E***J. thaiana* Leelawathanagoon, Lheknim & Ng, 2005, paratype male (22.2 × 18.6 mm) (ZRC 2006.0052), Thailand **F***J. booliati* sp. nov., holotype male (34.6 × 30.0 mm) (ZRC 2020.0072), Pahang **G***J. erici* sp. nov., holotype male (38.3 × 32.0 mm) (ZRC 2020.0360), Perak **H***J. michaeli* sp. nov., holotype male (22.7 × 19.2 mm) (ZRC 2010.0047), Terengganu.

Adult carapace width to length ratio 1.16–1.19 (Figs 1B, 2B, 12B); dorsal surface gently convex in frontal view, not inflated (Fig. 3B); frontal margin almost straight (Fig. 2B); suborbital, pterygostomial and sub-branchial regions rugose, pterygostomial region covered with dense setae (Fig. 3B); epigastric cristae distinct, distinctly anterior to sharp postorbital cristae, postorbital cristae with lateral edges low, joining lateral margin through oblique striae (Fig. 2B); external orbital tooth separated from epibranchial tooth by distinct cleft, epibranchial tooth sharp, distinct (Fig. 2B); anterolateral margin distinctly convex (Fig. 2B); posterolateral margin gently sinuous to almost straight, distinctly converging towards gently convex, entire posterior carapace margin (Fig. 2B); posterior margin of epistome with triangular median triangle, lateral margin obliquely sloping (Fig. 3B); outer surfaces of third maxillipeds with dense, long stiff setae; ischium subrectangular, with shallow median oblique groove (Figs 3B, 4B); ambulatory legs not elongate, length to width ratio of merus of fourth ambulatory leg 2.7–2.8 (Figs 1B, 12B); G1 subterminal segment gradually tapering from broad proximal part to slender distal part, without distinct shelf-like structure along gently concave outer margin; terminal segment gently curved outwards (from median part of sternum), ca. half length of subterminal segment, surface with numerous short setae (Fig. 8A–C); G2 slightly longer than G1, distal segment long, about half length of basal segment (Fig. 8D). Female pleon longitudinally ovate; somites 3–6 progressively narrower; telson subtriangular (Fig. 13B). Vulvae large, on anterior half of sternite 6, slightly pushing into suture with sternite 5, lateral sternal vulvar cover semicircular (Fig. 14B).

###### Description of male.

Carapace subrectangular broader than long, width to length ratio 1.16–1.19; dorsal surface gently convex in frontal view (Figs 1B, 2B, 3B). Frontal margin almost straight; frontal region, dorsal surface, lateral parts of anterolateral and branchial regions rugose, covered with small granules and striae; regions clearly indicated, median H-shaped gastro-cardiac groove deep; cervical grooves broad, shallow; suborbital, pterygostomial and sub-branchial regions rugose, covered with dense setae (Figs 2B, 3B). Epigastric cristae distinct, marked by transverse striae, not cristate, separated by median groove; postorbital cristae sharp, prominent, positioned distinctly posterior to and separated from epigastric cristae, lateral edges reaching beginning of cervical groove, reaching lateral margin through series of short, oblique striae (Figs 1B, 2B). Frontal margin entire; separated from supraorbital margin by rounded angle (Fig. 2B). External orbital tooth triangular, outer margin twice length of inner margin; epibranchial tooth sharp, distinct, separated from anterolateral margin by distinct cleft (Figs 1B, 2B). Anterolateral margins convex, cristate, granulated (Fig. 2B). Posterolateral margin gently sinuous to almost straight, distinctly converging towards gently convex, entire posterior carapace margin (Figs 1B, 2B). Orbits subovate; eye filling up most of orbital space; ocular peduncle relatively short, stout; cornea large, pigmented (Fig. 3B). Supraorbital margin concave, entire (Fig. 2B). Suborbital margin concave, complete, cristate (Fig. 3B). Antennules short, folding transversely in narrow fossa; antennae very short, not reaching cornea of eyes (Fig. 3B). Posterior margin of epistome with triangular median lobe; lateral part obliquely sloping, with 2 distinct concave margins (Fig. 3B). Mandibular palp with 3 distinct articles, terminal article single lobed.

Third maxillipeds covering most of buccal cavity when closed; surfaces of merus, ischium and exopod with numerous long stiff setae; ischium subrectangular, with shallow median oblique groove; merus subquadrate, slightly wider than long, anteroexternal angle not expanded; exopod slender, reaching half length of merus, with long flagellum (Figs 3B, 4B).

Chelipeds asymmetrical (Fig. 1B). Anterior margin of basis-ischium smooth; margins of merus rugose, uneven; inner margin lined with dense setae. Outer surface of carpus rugose, inner distal angle with sharp tooth (Fig. 1B). Outer surfaces of chelae rugose; chela stout (Fig. 1B). Fingers of chela almost straight, longer than palm; cutting edges of both fingers with variously sized teeth and denticles; fingers of slightly smaller chela similar (Fig. 1B).

Ambulatory legs not elongate, length to width ratio of merus of fourth ambulatory leg 2.7–2.8; second pair longest, fourth pair shortest (Fig. 1B). Surface of merus gently rugose, dorsal margin weakly cristate, slightly uneven, appears serrated, without subdistal spine or tooth; carpus rugose with short setae; propodus laterally flattened, margins with short setae; dactylus gently curved, setose, margins with short spines (Fig. 1B).

**Figure 5.**
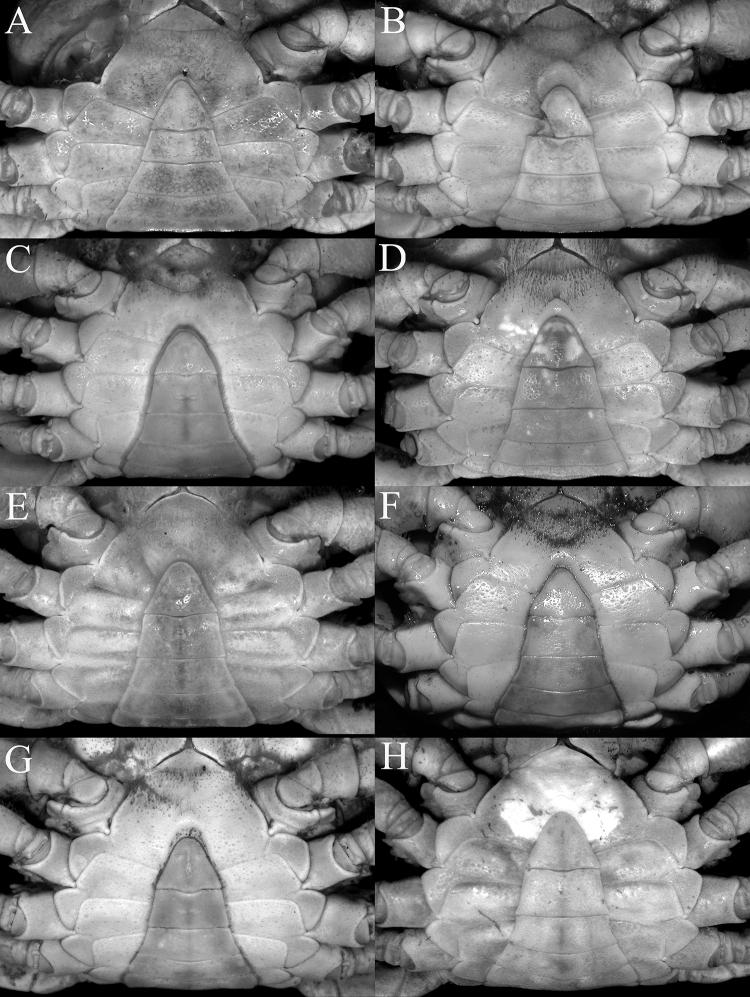
Anterior thoracic sternum and pleon **A***Johora intermedia* Ng, 1986, holotype male (19.8 × 15.1 mm) (ZRC 1984.6529), Pahang **B***J. tahanensis* (Bott, 1966), male (30.7 × 25.5 mm) (ZRC 1984.6795), Pahang **C***J. thoi* Ng, 1990, holotype male (41.2 × 33.3 mm) (ZRC 1989.2249), Terengganu **D***J. hoiseni* Ng & Takeda, 1992, holotype male (25.1 × 21.1 mm) (ZRC 1984.6673), Kelantan **E***J. thaiana* Leelawathanagoon, Lheknim & Ng, 2005, paratype male (22.2 × 18.6 mm) (ZRC 2006.0052), Thailand **F***J. booliati* sp. nov., holotype male (34.6 × 30.0 mm) (ZRC 2020.0072), Pahang **G***J. erici* sp. nov., holotype male (38.3 × 32.0 mm) (ZRC 2020.0360), Perak **H***J. michaeli* sp. nov., holotype male (22.7 × 19.2 mm) (ZRC 2010.0047), Terengganu.

Thoracic sternum (notably sternites 3, 4) with shallow pits to smooth (Fig. 5B). Sternites 1, 2 completely fused to form triangular plate with convex margins; separated from sternite 3 by distinct, gently concave suture lined with short setae; sternites 3, 4 completely fused except for shallow oblique depression between sternites, lined with short stiff setae (Fig. 5B); sutures between sternites 4/5, 5/6, 6/7 medially interrupted; suture between sternites 7, 8 complete; deep longitudinal groove on sternite 8 extending to most of sternite 7. Penis on condyle of coxa of fourth ambulatory leg. Sternopleonal cavity deep, reaching to imaginary line connecting submedian parts of cheliped coxae (Fig. 5B). Male pleonal locking tubercle relatively large, low, round, positioned on posterior edge of sternite 5, just adjacent to sternite 6.

Pleon triangular, all somites, telson free; telson triangular, lateral margins almost straight to gently sinuous; somite 6 subtrapezoidal, distinctly wider than long, lateral margins gently sinuous; somites 3–5 trapezoidal, gradually decreasing in width; somites 1, 2 subrectangular, narrow, very wide, reaching to bases of coxae of fourth ambulatory legs, thoracic sternite 8 not visible when pleon closed (Fig. 5B).

G1 subterminal segment gradually tapering from broad proximal part to slender distal part, without distinct shelf-like structure along gently concave outer margin; terminal segment gently curved outwards (from median part of sternum), ca. half length of subterminal segment, surfaces with numerous short setae (Fig. 8A–C); G2 slightly longer than G1, distal segment long, about half length of basal segment (Fig. 8D).

**Female**. Similar to male in most non-sexual features; chelipeds symmetrical or only slightly asymmetrical (Fig. 12B). Pleon longitudinally ovate, covering most of thoracic sternal surface, all somites and telson free; somites 3–6 progressively narrower; telson subtriangular (Fig. 13B). Vulvae large, covering anterior half of sternite 6, slightly pushing into suture with sternite 5, lateral sternal vulvar cover semicircular (Fig. 14B).

###### Remarks.

Ng and Takeda (1992: 108) discussed the taxonomy of *J. tahanensis* at length, showing that the material from two parts of Taman Negara National Park in Malaysia belonged to two species and that the type series is mixed. Bott (1966, 1970) and Ng (1987, 1988) had regarded them as one taxon, incorrectly noting that the G1 structure was variable. The holotype of *J. tahanensis* was from Kuala Tahan in the state of Pahang, in the southern part of the national park, and the G1 of this specimen (in the Senckenberg Museum, Frankfurt) as well as the topotypic material we have from that location has a terminal segment which is gently curved (Fig. 8A). Material from the northern part of the park from the adjacent state of Kelantan had a G1 terminal segment that is straight (Fig. 8E). Ng and Takeda (1992) showed that the G1 differences of the Pahang and Kelantan specimens are consistent and the Kelantan material was referred to a separate species, *J. hoiseni*.

The material reported as “*J. tahanensis*” by Ng and Takeda (1992: 108) from and around Fraser’s Hill in Selangor and Pahang should be now referred to *J. booliati* sp. nov. (see remarks for this species).

###### Distribution.

*Johora tahanensis* is known from the tributaries around Sungei Tahan in southern Pahang, at the southern part of Taman Negara National Park (Fig. 15). It lives among rocks and submerged vegetation in the relatively fast flowing streams in the forest.

###### Conservation.

The species is not under any immediate threat as it is found in Malaysia’s oldest and largest national park. Ng and Yeo (2007) treated *J. tahanensis* s. str. as vulnerable as it is not known from a relatively wide geographic area but it was reappraised and regarded as of least concern in Cumberlidge et al. (2009).

##### Johora thoi

Taxon classificationAnimaliaDecapodaPotamidae

Ng, 1990

EF6BEDB2-6B88-5D0D-9F4C-B693F42CC32C

Figures 1C , 2C , 3C , 4C , 5C , 6 , 9A–H , 12C , 13C , 14C

Johora thoi Ng, 1990: 305, figs 1, 2; Ng 2004: 321; Ng and Yeo 2007: 102; Ng et al. 2008: 163; Cumberlidge et al. 2009: table.

###### Material examined.

***Holotype***: male (41.2 × 33.3 mm) (ZRC 1989.2249), Telok Kalong Besar, Pulau Redang, Terengganu, ca. 5°46'7"N, 103°01'38"E, coll. Tho YP, 8 March 1989. Others: 1 male (ZRC 1989.3758), in freshwater stream, Telok Kalong Besar, Pulau Redang, Terengganu, coll. Saw LG, 1 August 1989; 1 male (ZRC 1989.3740), Telok Kalong Besar, Pulau Redang, Terengganu, coll. Saw LG, 1 August 1989; 1 male, 1 female (ZRC 1996.2085), site 5, stream behind Pasir Panjang, Pulau Redang, Terengganu, Lim KKP et al., 25 June 1992; 1 male (ZRC 1996.2087), Pulau Redang, Terengganu, Lim KKP et al., 23 June 1992; 3 males (ZRC 1996.2086), site 7, stream behind Telok Kalong Besar, Pulau Redang, Terengganu, coll. Lim KKP et al., 25 June 1992; 6 males, 2 females, 1 juvenile (ZRC 2001.1167), from freshwater rocky stream, East Coast forest trail, ca. 1.2–1.6 km from Pasir Panjang to Telok Dalam, Pulau Redang, Terengganu, coll. Tan HH and Koh LL, 20 June 2001. All locations in Peninsular Malaysia.

###### Diagnosis.

**Figure 6.**
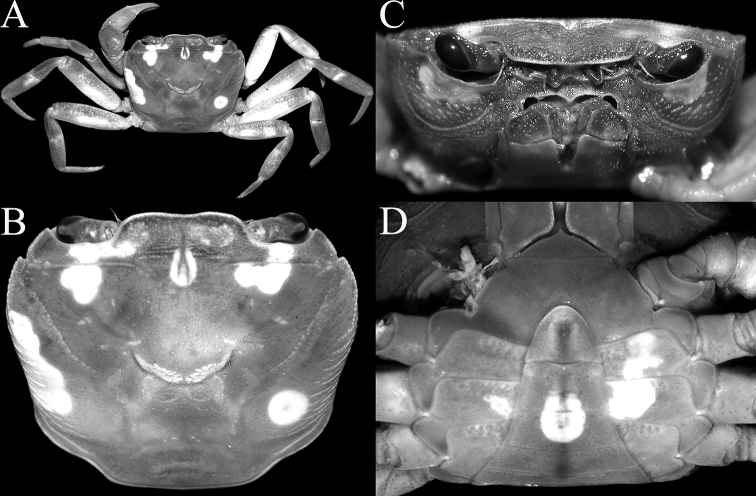
*Johora thoi* Ng, 1990, male (21.1 × 17.7 mm) (ZRC 2001.1167), Terengganu **A** overall dorsal view **B** dorsal view of carapace **C** frontal view of cephalothorax **D** anterior thoracic sternum and pleon.

Adult carapace width to length ratio 1.19–1.24 (Figs 1C, 2C, 6A, B, 12C); dorsal surface gently convex in frontal view, not inflated (Figs 3C, 6C); frontal margin almost straight or slightly sinuous (Figs 2C, 6B); suborbital, pterygostomial and sub-branchial regions rugose, pterygostomial region covered with dense setae (Figs 3C, 6C); epigastric cristae distinct, distinctly anterior to sharp postorbital cristae, postorbital cristae with lateral edges low, not joining lateral margin (Figs 2C, 6B); external orbital tooth separated from epibranchial tooth by prominent cleft, epibranchial tooth sharp, distinct (Figs 2C, 6B); anterolateral margin distinctly convex (Figs 2C, 6B); posterolateral margin gently sinuous to almost straight, distinctly converging towards gently convex, entire posterior carapace margin (Figs 2C, 6B); posterior margin of epistome with triangular median triangle, lateral margin obliquely sloping (Figs 3C, 6C); outer surfaces of third maxillipeds with dense, long stiff setae; ischium subrectangular, with shallow median oblique groove (Figs 3C, 4C); ambulatory legs relatively longer, length to width ratio of merus of fourth ambulatory leg 3.0–3.2 (Figs 1C, 6A, 12C); G1 subterminal segment with broad proximal part, tapering suddenly to slender distal part, with distinct shelf-like structure along outer margin; terminal segment straight, long, slender, subequal in length to subterminal segment, surfaces with scattered short setae (Fig. 9A–C, E–G); G2 shorter than G1, distal segment long, about two-thirds length of basal segment (Fig. 9D, H). Female pleon ovate; somites 3–6 progressively narrower; telson semi-circular (Fig. 13C). Vulvae large, on anterior half of sternite 6, adjacent to suture with sternite 5, lateral sternal vulvar cover triangular, partially overlapping sternite 5 (Fig. 14C).

**Figure 7.**
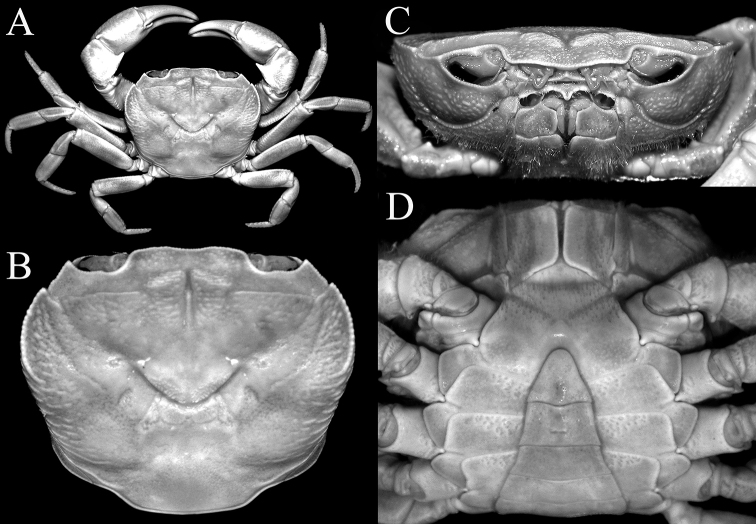
*Johora erici* sp. nov., male (41.2 × 33.8 mm) (ZRC 1995.0269), Kelantan **A** overall dorsal view **B** dorsal view of carapace **C** frontal view of cephalothorax **D** anterior thoracic sternum and pleon.

###### Remarks.

This is one of the largest species of *Johora* and is rivalled in size only by *J. tiomanensis* and *J. counsilmani* from Pulau Tioman. The G1 structure of *J. thoi* is distinctive and consistent, the long and straight terminal segment being evident even in small subadult specimens (Fig. 9E–G). The only other species with superficially similar G1s are *J. singaporensis* and *J. michaeli* sp. nov. from Singapore and mainland Terengganu, respectively. In these species, however, the G1 terminal segment is prominently shorter, being only two-thirds or less the length of the subterminal segment (Fig. 9I–K; Ng 1987: fig. 8A, B).

**Figure 8.**
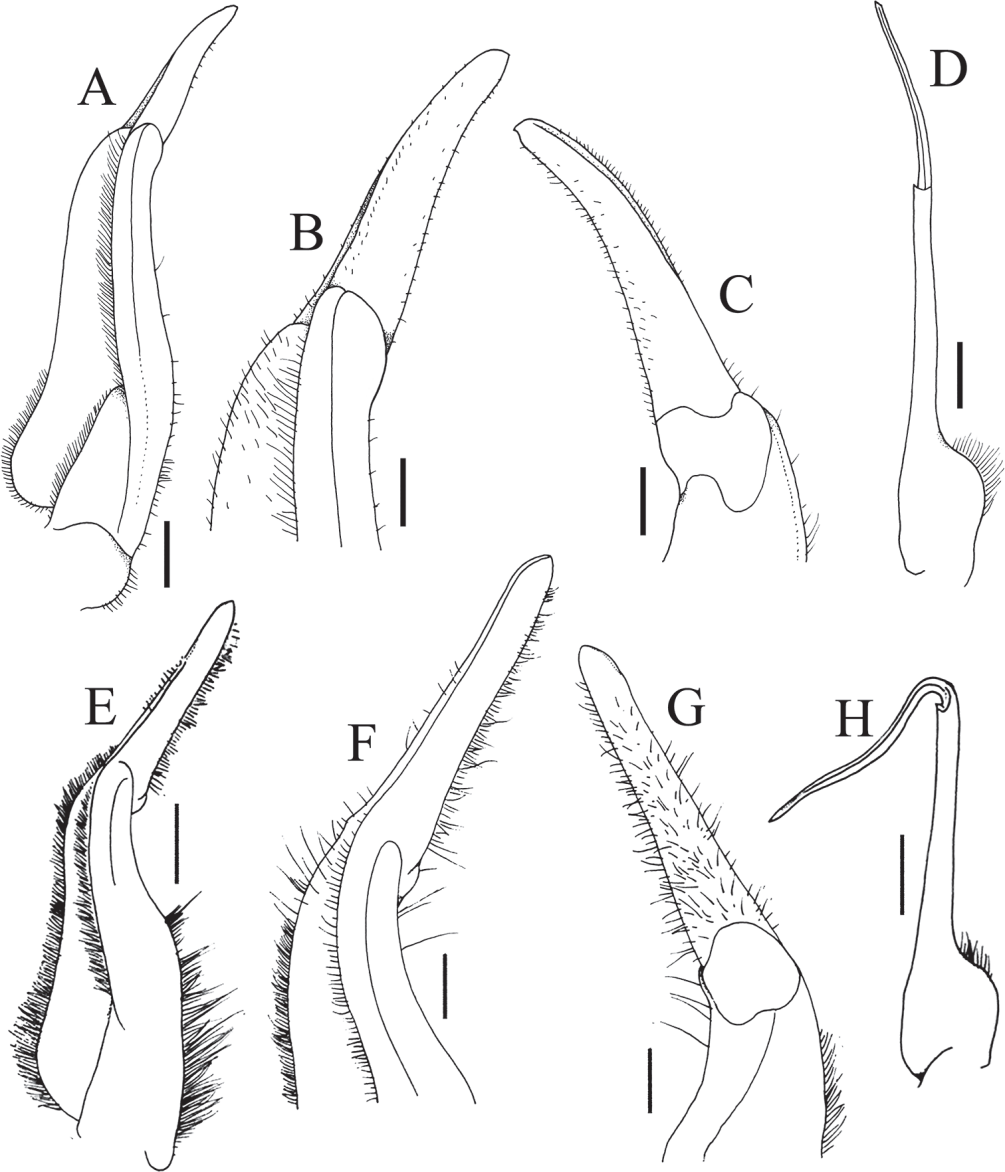
**A–D***Johora tahanensis* (Bott, 1966), male (30.7 × 25.5 mm) (ZRC 1984.6795), Pahang **E–H***J. hoiseni* Ng & Takeda, 1992, holotype male (25.1 × 21.1 mm) (ZRC 1984.6673), Kelantan **A, E** left G1 (ventral view) **B, F** distal part of left G1 (ventral view) **C, G** distal part of left G1 (dorsal view) **D, H** left G2 (ventral view) **E–H** after Ng and Takeda (1992: fig. 3B, D–F). Scale bars: 1.0 mm (**A, D, E, H**); 0.5 mm (**B, C, F, G**).

**Figure 9.**
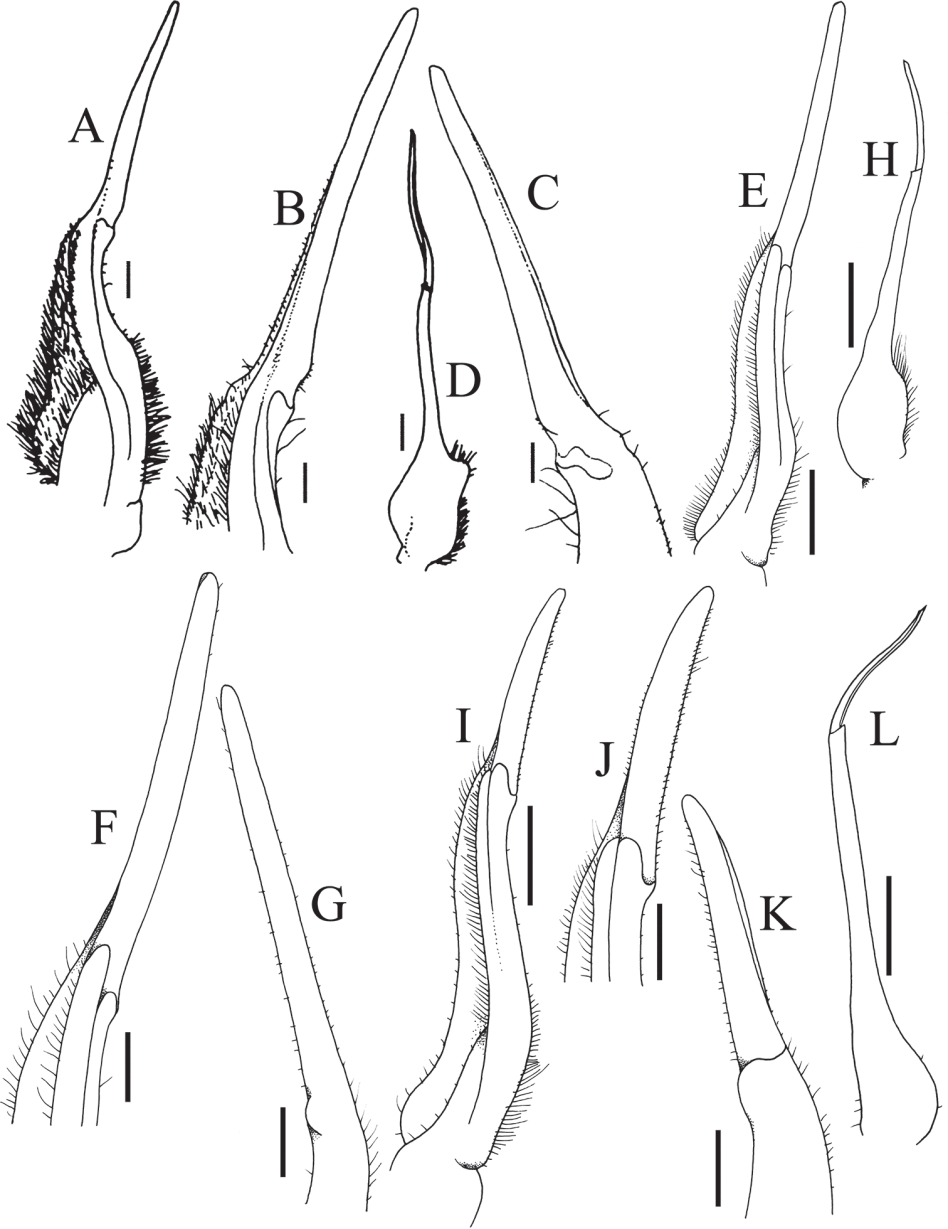
**A–D***Johora thoi* Ng, 1990, holotype male (41.2 × 33.3 mm) (ZRC 1989.2249), Terengganu **E–H***J. thoi* Ng, 1990, male (21.1 × 17.7 mm) (ZRC 2001.1167), Terengganu **I–L***J. michaeli* sp. nov., holotype male (22.7 × 19.2 mm) (ZRC 2020.0361), Terengganu **A, E, I** left G1 (ventral view) **B, F, J** distal part of left G1 (ventral view) **C, G, K** distal part of left G1 (dorsal view) **D, H, L** left G2 (ventral view) **A–D** after Ng (1990: fig. 2A, C–E). Scale bars: 1.0 mm (**A, D, E, H, I, L**); 0.5 mm (**B, C, F, G, J, K)**.

###### Distribution.

*Johora thoi* is endemic to Pulau Redang, the largest island in the Redang Archipelago, a group of nine islands about 25 km off the northeastern coast of Peninsular Malaysia. The species has been recorded from most parts of the island, including its highest point (359 m), occurring in all clean waters there (Fig. 15). It lives under rocks and can be found along the stream banks at night.

###### Conservation.

Although the entire Redang Archipelago is a marine park, the forests are not fully protected, and development of the land for tourism and excessive freshwater use are concerns. As the species is only known from one island only 7 km long and 6 km wide, it is treated as endangered by Ng and Yeo (2007), but Cumberlidge et al. (2009) noted it was vulnerable at best as the area is technically protected.

##### Johora hoiseni

Taxon classificationAnimaliaDecapodaPotamidae

Ng & Takeda, 1992

20070E29-AF8A-5A3B-BDFB-F018D2BB4A2B

Figures 1D , 2D , 3D , 4D , 5D , 8E–H , 12D , 13D , 14D

Potamiscus (Johora) johorensis tahanensis Bott, 1966: 495 (part), fig. 32 (not Stoliczia (Johora) johorensis tahanensis Bott, 1966 s. str.).
Stoliczia (Johora) johorensis tahanensis – Bott 1970: 181 (part), pl. 40 fig. 57; Ng and Tan 1984: 172, fig. 7 (not Stoliczia (Johora) johorensis tahanensis Bott, 1966).
Johora tahanensis – Ng 1987: 33 (part), fig. 9B–K; Ng 1988: 42 (part), fig. 18A, B, D, E, G (not Stoliczia (Johora) johorensis tahanensis Bott, 1966).
Johora hoiseni Ng & Takeda, 1992: 108, figs 2, 3; Ng 2004: 321; Ng and Yeo 2007: 100; Ng et al. 2008: 163; Cumberlidge et al. 2009: table.

###### Material examined.

***Holotype***: male (25.1 × 21.1 mm) (ZRC 1984.6673), stream entering Sungei Galas, near Gua Madir, Taman Negara National Park, Kelantan, ca. 4°51'30"N, 102°03'23"E, coll. Tweedie MWF, August 1939. Paratypes: 53 males, 13 females (ZRC 1984.6674–6755), same data as holotype. Others: 3 males, 18 females (ZRC 1984.7683–7687), stream entering Sungei Galas, near Gua Madir, Taman Negara National Park, Kelantan, ca. 4°51'30"N, 102°03'23"E, coll. Tweedie MWF, August 1939; 2 males, 5 females (ZRC 1989.3617–3623), Pahang or Kelantan?, no other data. All locations in Peninsular Malaysia.

###### Diagnosis.

Adult carapace width to length ratio 1.16–1.19 (Figs 1D, 2D, 12D); dorsal surface gently convex in frontal view, not inflated (Fig. 3D); frontal margin slightly sinuous to almost straight (Fig. 2D); suborbital, pterygostomial and sub-branchial regions rugose, pterygostomial region covered with dense setae (Fig. 3D); epigastric cristae distinct, distinctly anterior to sharp postorbital cristae, postorbital cristae with lateral edges low, joining lateral margin through oblique striae (Fig. 2D); external orbital tooth separated from epibranchial tooth by distinct cleft, epibranchial tooth sharp, distinct (Fig. 2D); anterolateral margin gently convex (Fig. 2D); posterolateral margin medially concave to sinuous, gently converging towards gently convex, entire posterior carapace margin (Fig. 2D); posterior margin of epistome with triangular median triangle, lateral margin obliquely sloping (Fig. 3D); outer surfaces of third maxillipeds with dense, long stiff setae; ischium subrectangular, with shallow median oblique groove (Figs 3D, 4D); ambulatory legs not elongate, length to width ratio of merus of fourth ambulatory leg 2.7–2.8 (Figs 1D, 12D); G1 subterminal segment with broad proximal part, tapering suddenly to slender distal part, with distinct shelf-like structure along outer margin; terminal segment straight, ca. two-thirds length of subterminal segment, surfaces with numerous short setae (Fig. 8E–G); G2 slightly longer than G1, distal segment long, about half length of basal segment (Fig. 8H). Female pleon ovate; somite 3 less wide than somite 4, somites 4–6 progressively narrower; telson semicircular (Fig. 13D). Vulvae large, on anterior half of sternite 6, slightly pushing into suture with sternite 5, lateral sternal vulvar cover semicircular (Fig. 14D).

###### Remarks.

Ng and Takeda (1992) showed that the G1 differences of the material that had been identified as “*J. tahanensis*” by Bott (1966, 1970; Ng 1987, 1988) belong to a distinct species, *J. hoiseni* (see remarks for *J. tahanensis*).

###### Distribution.

*Johora hoiseni* is known from the drainages near Gua Musang in Kelantan, in the northwestern part of Taman Negara National Park (Fig. 15).

###### Conservation.

The species is not under any immediate threat as it is found in Malaysia’s oldest and largest national park. Ng and Yeo (2007) treated the species as endangered as it is only known from a relatively small geographic area but Cumberlidge et al. (2009) listed it as of least concern as it is in a protected area.

##### Johora thaiana

Taxon classificationAnimaliaDecapodaPotamidae

Leelawathanagoon, Lheknim & Ng, 2005

9CE1056C-862D-5E29-9822-55A0E22BFC13

Figures 1E , 2E , 3E , 4E , 5E , 10A–D , 12E , 13E , 14E

Johora thaiana Leelawathanagoon, Lheknim & Ng, 2005: 60, figs 1, 2; Cumberlidge et al. 2009: table.

###### Material examined.

***Paratypes***: 1 male (22.2 × 18.6 mm) (ZRC 2006.0052), Huai Sam Sop, Ko Lok River Basin, Ban Ba La, 140 m above sea level, 5.71583°N, 101.83917°E, Wang District, Narathiwat Province, southern Thailand, coll. Lheknim V, 14 July 1999; 1 female (21.0 × 17.4 mm) (ZRC 2006.0053), Sirindthron Waterfall, Ban Ba La, 300 m above sea level, 5.8°N, 101.82083°E, Wang District, southern Thailand, coll. Lheknim V, 15 July 1999.

###### Diagnosis.

**Figure 10.**
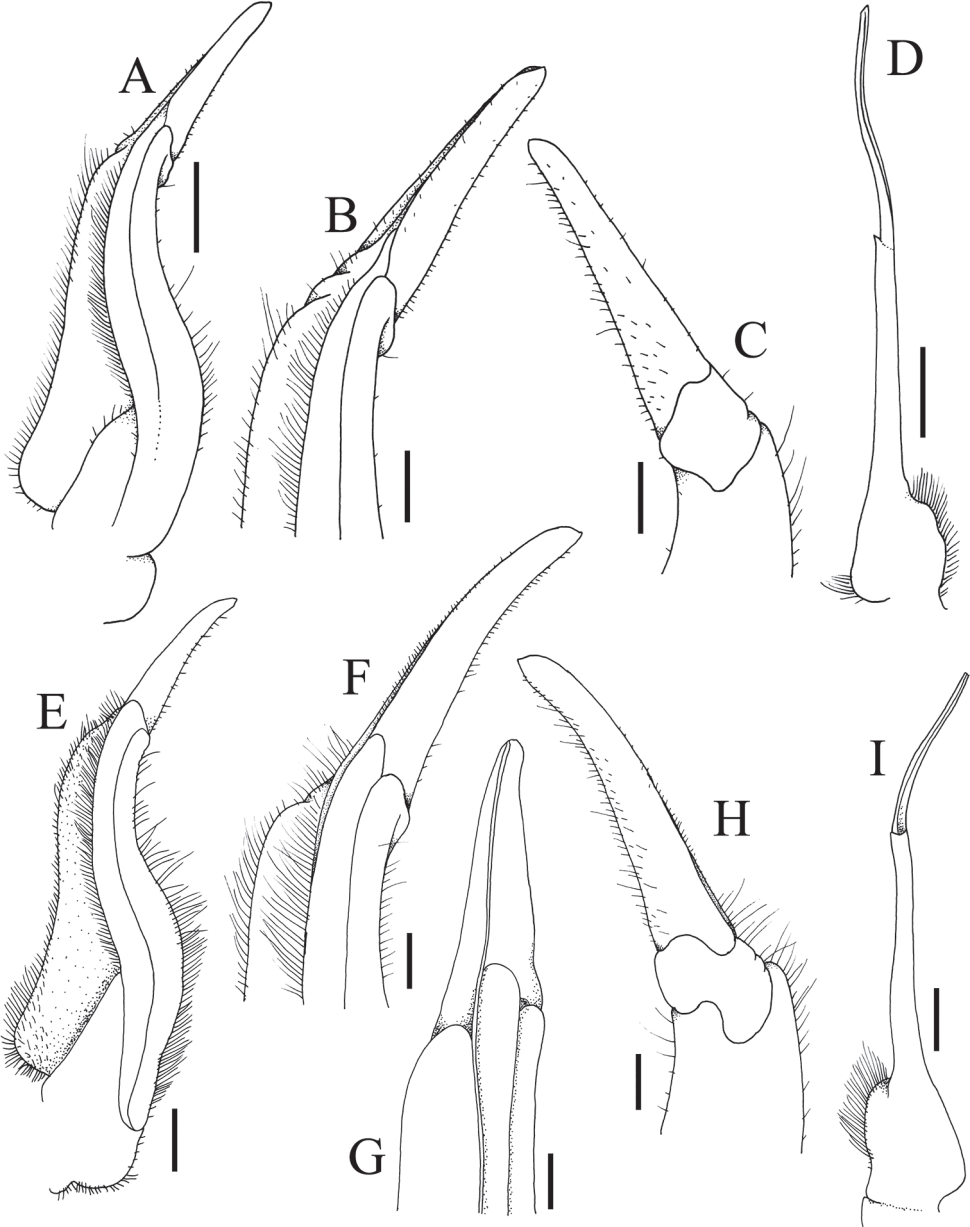
**A–D***Johora thaiana* Leelawathanagoon, Lheknim & Ng, 2005, paratype male (22.2 × 18.6 mm) (ZRC 2006.0052), Thailand **E–I***J. booliati* sp. nov., holotype male (34.6 × 30.0 mm) (ZRC 2020.0072), Pahang **A, E** left G1 (ventral view) **B, F** distal part of left G1 (ventral view) **C, H** distal part of left G1 (dorsal view) **G** distal part of left G1 (mesial view) **D, I** left G2 (ventral view). Scale bas: 1.0 mm (**A, D, E, I**); 0.5 mm (**B, C, F–H**).

Adult carapace width to length ratio 1.10–1.21 (Figs 1E, 2E, 12E); dorsal surface gently convex in frontal view, not inflated (Fig. 3E); frontal margin almost straight (Fig. 2E); suborbital, pterygostomial and sub-branchial regions rugose, pterygostomial region covered with dense setae (Fig. 3E); epigastric cristae distinct, distinctly anterior to sharp postorbital cristae, postorbital cristae with lateral edges low, not joining lateral margin (Fig. 2E); external orbital tooth separated from epibranchial tooth by distinct cleft, epibranchial tooth sharp, distinct (Fig. 2E); anterolateral margin distinctly convex (Fig. 2E); posterolateral margin medially concave, distinctly converging towards gently convex, entire posterior carapace margin (Fig. 2E); posterior margin of epistome with triangular median triangle, lateral margin obliquely sloping (Fig. 3E); outer surfaces of third maxillipeds with dense, long stiff setae; ischium subrectangular, with shallow median oblique groove (Figs 3E, 4E); ambulatory legs not elongate, length to width ratio of merus of fourth ambulatory leg 2.7–2.8 (Figs 1E, 12E); G1 subterminal segment gradually tapering from broad proximal part to slender distal part, without distinct shelf-like structure along gently concave outer margin; terminal segment almost straight, ca. half length of subterminal segment, surfaces with numerous short setae (Fig. 10A–C); G2 longer than G1, distal segment long, about two-thirds length of basal segment (Fig. 10D). Female pleon ovate; somites 3–6 progressively narrower; telson semicircular (Fig. 13E). Vulvae large, on anterior half of sternite 6, slightly pushing into suture with sternite 5, lateral sternal vulvar cover semicircular (Fig. 14E).

###### Remarks.

**Figure 11.**
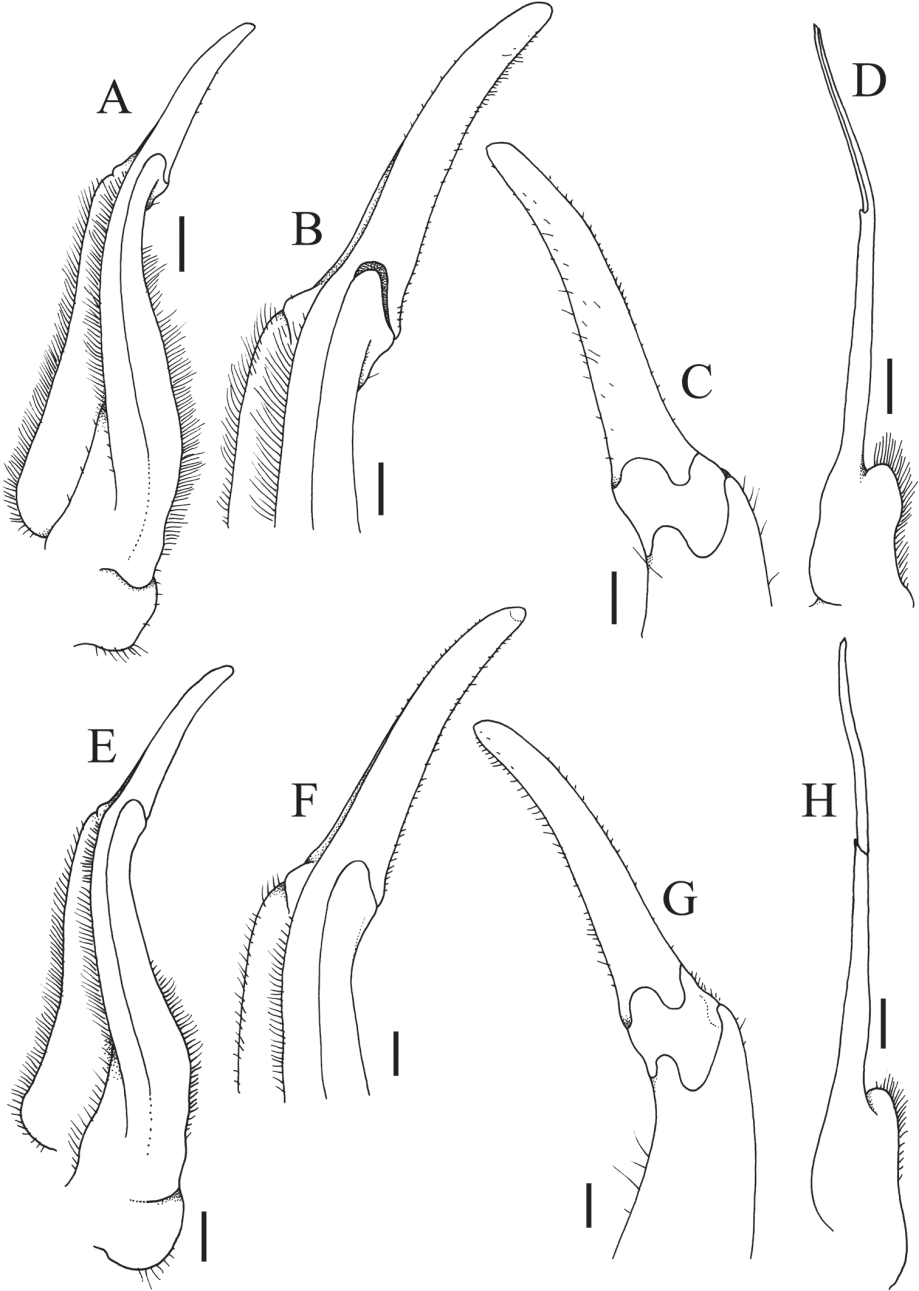
**A–D***Johora erici* sp. nov., holotype male (38.3 × 32.0 mm) (ZRC 2020.0360), Perak **E–H***J. erici* sp. nov., male (41.2 × 33.8 mm) (ZRC 1995.0269), Kelantan **A, E** left G1 (ventral view) **B, F** distal part of left G1 (ventral view) **C, G** distal part of left G1 (dorsal view) **D, H** left G2 (ventral view). Scale bars: 1.0 mm (**A, D, E, H**); 0.5 mm (**B, C, F, G**).

The G1 structure of *J. thaiana* most closely resembles that of *J. hoiseni* in that the terminal segment is straight; but their subterminal segments differ. In *J. thaiana*, the subterminal segment gradually tapers from the broad proximal part to a slender distal section, without a shelf-like structure along the outer margin (Fig. 10A); in *J. hoiseni*, the distal part of the subterminal segment becomes slender more abruptly, resulting in distinct shelf-like structure on the outer margin (Fig. 8E). Biogeographically, the type localities of *J. thaiana* are less than 20 km from the *J. erici* sp. nov. in Jeli, Kelantan; but their carapace features and G1s differ markedly (Figs 2E, 10A–C versus Figs 2G, 7B, 11A–C, E–G).

###### Distribution.

*Johora thaiana* is known from the forest tributaries in southern Thailand, near the Malaysian border at Kelantan (Fig. 15). Its distribution is adjacent to that of *J. erici* sp. nov. which lives in the highlands of northern Malaysia.

###### Conservation.

The species has so far only been found in protected forests in southern Thailand and is not under any immediate threat; Cumberlidge et al. (2009) list it as of least concern.

##### Johora booliati sp. nov.

Taxon classificationAnimaliaDecapodaPotamidae

253627CA-174B-5C69-B8B6-231C187A0709

http://zoobank.org/8A3C9173-A7A5-4559-92D7-A548453C6688

Figures 1F , 2F , 3F , 4F , 5F , 10E–I , 12F , 13F , 14F

Johora tahanensis – Takeda & Ng, 1992: 107 (part); Ng 1995: 249 (part) (not Stoliczia (Johora) johorensis tahanensis Bott, 1966).

###### Material examined.

***Holotype***: male (34.6 × 30.0 mm) (ZRC 2020.0072), stream next to house, under rocks, in old farmland and secondary forest, Bukit Tinggi village, west side of northbound Karak Highway, ca. 480 m a.s.l., Bentong District, Pahang, 3°22'3.0396"N, 101°48'50.994"E, coll. July 2016. Paratype: 1 female (40.4 × 34.4 mm) (ZRC 1995.270), in shallow stream with leaf litter, The Gap, Fraser’s Hill, 853 m a.s.l., Pahang, ca. 3°41'29"N, 101°44'56"E, coll. Lim KKP, 1 June 1990; 1 juvenile male (10.5 × 9.1 mm) (ZRC 2020.0364), under rocks, first stream on road towards Raub, descending from Fraser’s Hill, Pahang, coll. Lai JCY, 6 December 2015. All locations in Peninsular Malaysia.

###### Diagnosis.

**Figure 12.**
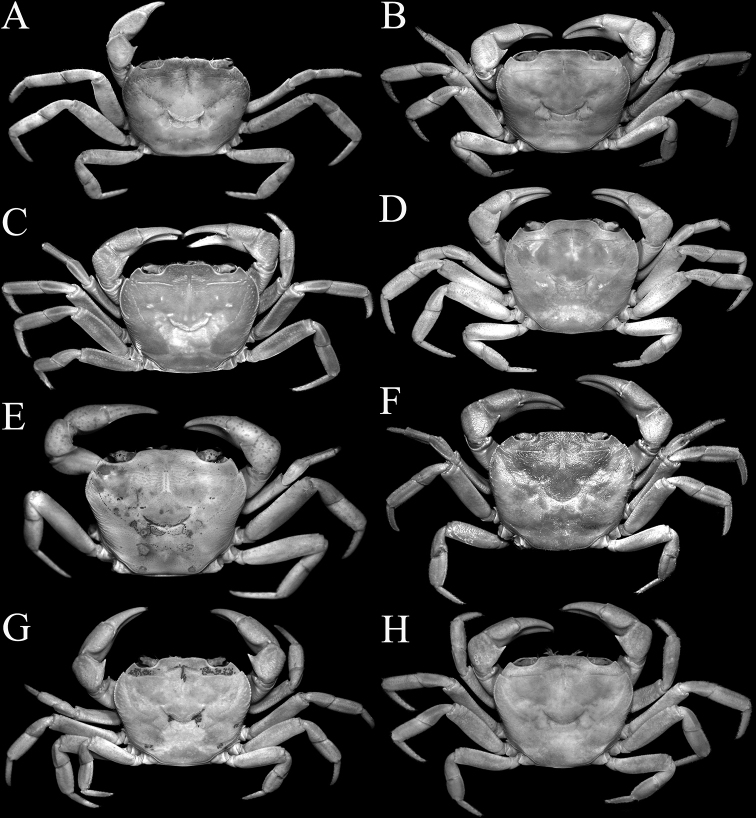
Overall dorsal view **A***Johora intermedia* Ng, 1986, female (17.9 × 14.7 mm) (ZRC 2001.1002), Selangor **B***J. tahanensis* (Bott, 1966), female (24.4 × 21.0 mm) (ZRC 1984.6765), Pahang **C***J. thoi* Ng, 1990, female (31.7 × 25.5 mm) (ZRC 2001.1167), Terengganu **D***J. hoiseni* Ng & Takeda, 1992, paratype female (22.1 × 19.0 mm) (ZRC 1984.6675), Kelantan **E***J. thaiana* Leelawathanagoon, Lheknim & Ng, 2005, paratype female (21.0 × 17.4 mm) (ZRC 2006.0053), Thailand **F***J. booliati* sp. nov., paratype female (40.4 × 34.4 mm) (ZRC 1995.270), Pahang **G***J. erici* sp. nov., paratype female (32.8 × 26.9 mm) (ZRC 1995.268), Perak **H***J. michaeli* sp. nov., paratype female (29.9 × 25.0 mm) (ZRC 2010.0047), Terengganu.

**Figure 13.**
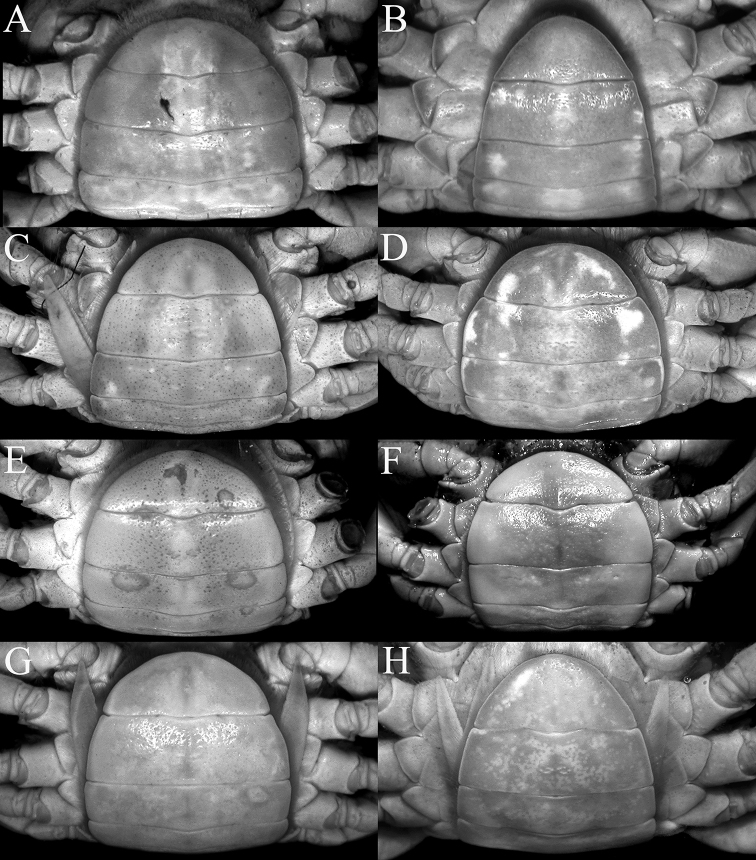
Female pleon **A***Johora intermedia* Ng, 1986, female (17.9 × 14.7 mm) (ZRC 2001.1002), Selangor **B***J. tahanensis* (Bott, 1966), female (24.4 × 21.0 mm) (ZRC 1984.6765), Pahang **C***J. thoi* Ng, 1990, female (31.7 × 25.5 mm) (ZRC 2001.1167), Terengganu **D***J. hoiseni* Ng & Takeda, 1992, paratype female (22.1 × 19.0 mm) (ZRC 1984.6675), Kelantan **E***J. thaiana* Leelawathanagoon, Lheknim & Ng, 2005, paratype female (21.0 × 17.4 mm) (ZRC 2006.0053), Thailand **F***J. booliati* sp. nov., paratype female (40.4 × 34.4 mm) (ZRC 1995.270), Pahang **G***J. erici* sp. nov., paratype female (32.8 × 26.9 mm) (ZRC 1995.268), Perak **H***J. michaeli* sp. nov., paratype female (29.9 × 25.0 mm) (ZRC 2010.0047), Terengganu.

**Figure 14.**
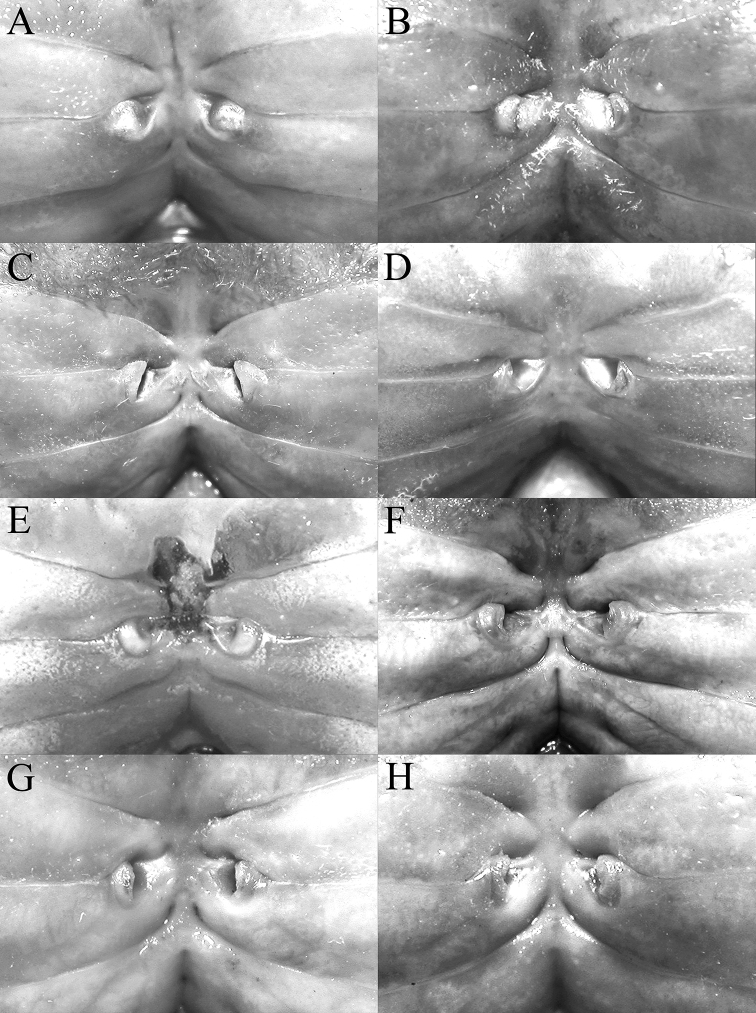
Sternopleonal cavity and vulvae **A***Johora intermedia* Ng, 1986, female (17.9 × 14.7 mm) (ZRC 2001.1002), Selangor **B***J. tahanensis* (Bott, 1966), female (24.4 × 21.0 mm) (ZRC 1984.6765), Pahang **C***J. thoi* Ng, 1990, female (31.7 × 25.5 mm) (ZRC 2001.1167), Terengganu **D***J. hoiseni* Ng & Takeda, 1992, paratype female (22.1 × 19.0 mm) (ZRC 1984.6675), Kelantan **E***J. thaiana* Leelawathanagoon, Lheknim & Ng, 2005, paratype female (21.0 × 17.4 mm) (ZRC 2006.0053), Thailand **F***J. booliati* sp. nov., paratype female (40.4 × 34.4 mm) (ZRC 1995.270), Pahang **G***J. erici* sp. nov., paratype female (32.8 × 26.9 mm) (ZRC 1995.268), Perak **H***J. michaeli* sp. nov., paratype female (29.9 × 25.0 mm) (ZRC 2010.0047), Terengganu.

Adult carapace width to length ratio 1.15–1.17 (Figs 1F, 2F, 12F); dorsal surface gently convex in frontal view, not inflated (Fig. 3F); frontal margin almost straight (Fig. 2F); suborbital, pterygostomial and sub-branchial regions rugose, pterygostomial region covered with dense setae (Fig. 3F); epigastric cristae distinct, distinctly anterior to sharp postorbital cristae, postorbital cristae with lateral edges low, joining lateral margin through oblique striae (Fig. 2F); external orbital tooth separated from epibranchial tooth by distinct cleft, epibranchial tooth sharp, distinct (Fig. 2F); anterolateral margin gently convex (Fig. 2F); posterolateral margin gently sinuous to almost straight, gently converging towards sinuous posterior carapace margin with shallow median indentation (Fig. 2F); posterior margin of epistome with triangular median triangle, lateral margin obliquely sloping (Fig. 3F); outer surfaces of third maxillipeds with dense, long stiff setae; ischium subrectangular, with shallow median oblique groove (Figs 3F, 4F); ambulatory legs not elongate, length to width ratio of merus of fourth ambulatory leg 2.7–2.8 (Figs 1F, 12F); G1 subterminal segment with broad proximal part, tapering relatively suddenly to slender distal part, with low shelf-like structure on outer margin; terminal segment gently curved outwards (from median part of sternum), less than half length of subterminal segment, surfaces with numerous short setae (Fig. 10E–H); G2 slightly longer than G1, distal segment long, about half length of basal segment (Fig. 10I). Female pleon ovate; somite 3 less wide than somite 4, somites 4–6 progressively narrower; telson semi-circular (Fig. 13F). Vulvae large, on anterior half of sternite 6, slightly pushing into suture with sternite 5, lateral sternal vulvar cover semi-circular (Fig. 14F).

###### Etymology.

The species is named after an old friend and mentor, the late Dr Lim Boo Liat (1926–2020), one of Malaysia’s most eminent biologists and naturalists. In a remarkable career spanning 70 years, he has contributed immensely to biodiversity and conservation science in Malaysia; more importantly, he has inspired, mentored, and trained many generations of biologists, many who now lead science in Malaysia and Singapore.

###### Remarks.

The large adult size of *J. booliati* sp. nov. and *J. erici* sp. nov. (carapace width in excess of 25 mm) with the pronounced epibranchial teeth allies them with *J. tahanensis* s. str., *J. hoiseni*, *J. thaiana*, *J. thoi*, *J. tiomanensis*, and *J. counsilmani* (the last two being endemic to Pulau Tioman, Peninsular Malaysia).

*Johora booliati* and *J. erici* resemble *J. tahanensis*, *J. hoiseni*, and *J. thaiana* in the carapace shape and general features. Their G1 structures, however, are different. In *J. booliati* and *J. erici*, the G1 subterminal segment is proportionately more elongate and the terminal segment is relatively shorter (Figs 10E–H, 11A–C, E–G), and significantly, the terminal segment has only scattered short setae (versus subterminal segment shorter with the terminal segment longer and the surface of the latter more densely covered with short setae in *J. tahanensis* (Fig. 8A–C). Both *J. hoiseni* and *J. thaiana* have relatively straight G1 terminal segments which are prominently setose (Figs 8E–G, 10A–C).

*Johora booliati* and *J. erici* can be distinguished from *J. tiomanensis* and *J. counsilmani* by their carapace being relatively more quadrate in shape (width to length ratio less than 1.2) and the adult carapace is distinctly wider than long (width to length ratio 1.3–1.4) in *J. tiomanensis* and *J. counsilmani*. In addition, the epigastric cristae of *J. booliati* and *J. erici* are distinctly anterior to the postorbital cristae and separated by a gap with the entire postorbital cristae sharp (Figs 2F, G, 7B) while in *J. tiomanensis* and *J. counsilmani*, the epigastric cristae are only slightly anterior of the postorbital cristae and separated only by striae (cf. Ng and Tan 1984: figs 1A, 2A; Ng 1985: fig. 3). Most significantly, the G1 terminal segments of *J. booliati* and *J. erici* are proportionately stouter and less curved (Figs 10E–H, 11A–C, E–G); in *J. tiomanensis* and *J. counsilmani*, it is distinctly slenderer, more elongate and strongly curved (cf. Ng and Tan 1984: fig. 3a–c; Ng 1985: figs 2a, c, e, 4a–c). *Johora booliati* and *J. erici* differ from *J. thoi* in possessing slightly shorter ambulatory legs (length to width ratio of merus of fourth leg 2.7–2.8 versus 3.0–3.2), the lateral carapace surfaces are relatively more rugose, and the distinctly shorter and gently curved G1 terminal segment (Figs 10E–H, 11A–C, E–G); in *J. thoi*, this structure is diagnostic, being very elongate and straight (Fig. 9A–C, E–G).

The G1 structure of *J. booliati* closely resembles that of *J. erici*, with the terminal segments similar in shape and proportions. The G1 subterminal segments of the two species, however, differ in form, with that of *J. booliati* forming a shelf-like structure along the outer margin (Fig. 10E) while in *J. erici*, the tapering of the segment is gradual, and no shelf is visible (Fig. 11A, E). The carapaces of the two species are different in adult males and females. In *J. booliati*, the carapace has a more quadrate form because the anterolateral margin is only gently convex, even in the largest male and the posterolateral margin is straighter and gently converging, with the posterior carapace margin medially indented (even in females) (Fig. 2F). In *J. erici*, the carapace appears more ovate, with the anterolateral margin distinctly convex, and the posterolateral margin is medially concave and strongly converging, and the posterior carapace margin is entire (Figs 2G, 7B).

The G1 structure of *J. booliati* and *J. erici* is superficially similar to that of *J. murphyi* from southern Peninsular Malaysia, notably in the shape of the G1 terminal and subterminal segments (Ng 1986b: fig. 14a, b; 1987: fig. 6D, E; 1988: fig. 16D, E), but differs in having the distal part of the terminal segment more rounded and less sharply tapering (Figs 10E–H, 11A–C, E–G). The G1 of *J. booliati* is also similar to *J. intermedia*, which has a wide distribution in central Peninsular Malaysia. The G1 terminal segment of *J. booliati* and *J. erici*, however, is relatively stouter and less curved, and the subterminal segment narrows along the distal third to form a neck-like structure with the terminal segment (Figs 10E–H, 11A–C, E–G) (versus the G1 terminal segment is more curved and tapers to a slender tip with only the distalmost part of the subterminal segment distinctly narrowed to form a broad cleft in *J. intermedia*, cf. Ng 1987: figs 3A–D, G–J, 4A–D, F, G, I, J; 1988: fig. 15D–F; Ng and Takeda 1992: fig. 1A–E). It is also noteworthy that both *J. murphyi* and *J. intermedia* are smaller species than *J. booliati* and *J. erici*, with adults not reaching carapace widths of 25 mm.

Ng and Takeda (1992: 107) had specimens from Fraser’s Hill as well as adjacent areas (Sungei Gumut, Peretak, Selangor, 3°36'53.3"N, 101°44'40.4"E; Sungei Sum [probably Sungei Sum Sum], near Genting Highlands, Pahang, 3°20'42.2"N, 101°51'12.0"E; Ulu Teranum, Teras, Pahang, 3°44'12.6"N, 101°47'29.5"E) which they identified as “*J. tahanensis*”. On the basis of geography, they are probably all *J. booliati* as defined here.

###### Distribution.

**Figure 15.**
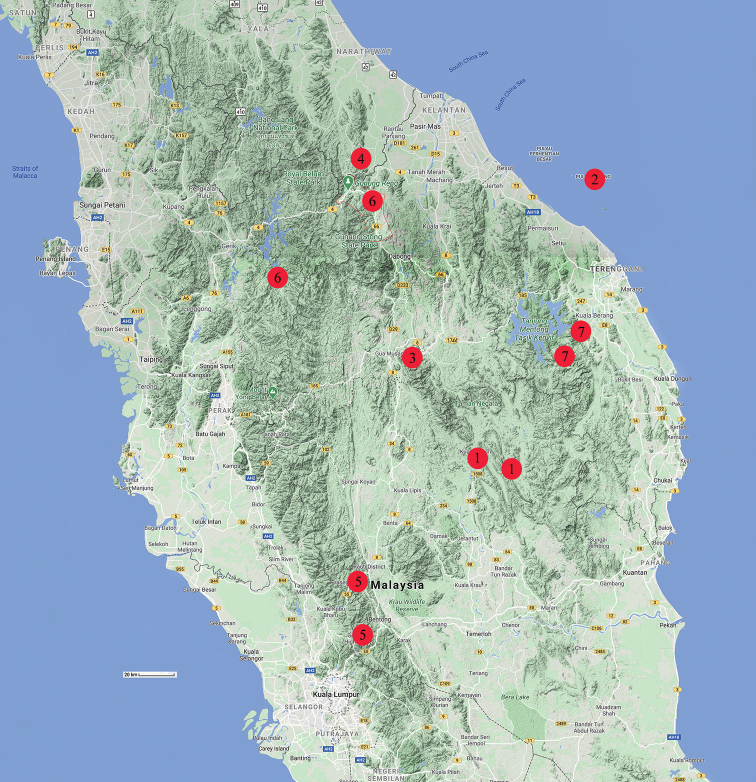
Distribution of species of the *Johora tahanensis* species group **1***J. tahanensis* (Bott, 1966) **2***J. thoi* Ng, 1990 **3***J. hoiseni* Ng & Takeda, 1992 **4***J. thaiana* Leelawathanagoon, Lheknim & Ng, 2005 **5***J. booliati* sp. nov. **6***J. erici* sp. nov. **7***J. michaeli* sp. nov. Map data 2020 Google.

*Johora booliati* sp. nov. is known from highland streams in the central highlands of Pahang (Genting Highlands, Bukit Tinggi and Fraser’s Hill) (Fig. 15). The distribution of *J. booliati* overlaps with those of *J. intermedia* and *J. gapensis*, and we can expect the taxa to be found together. One juvenile male of *J. booliati* (10.5 × 9.1 mm, ZRC 2020.0364) was in fact collected with two adult males of *J. intermedia* at Fraser’s Hill (ZRC 2016.003).

###### Conservation.

The conservation status for *J. booliati* is not known as its actual distribution is not known. For the moment, it is known only from a 30 km stretch of hills along the Central Highlands of Peninsular Malaysia, in the area of Fraser’s Hill, Genting Highlands and Bukit Tinggi. This area is not protected, and in this context, the species should be categorised as vulnerable for the time being (see Cumberlidge et al. 2009).

##### Johora erici sp. nov.

Taxon classificationAnimaliaDecapodaPotamidae

87868EFD-9CBA-5DE8-826C-D377FD475EE7

http://zoobank.org/881E59DF-ACD2-4E4B-91A1-F9CE77725965

Figures 1G , 2G , 3G , 4G , 5G , 7 , 11 , 12G , 13G , 14G

Johora tahanensis – Ng, 1995: 249 (part), fig. 1; Yeo et al. 2007: 257 (not Stoliczia (Johora) johorensis tahanensis Bott, 1966).

###### Material examined.

***Holotype***: male (38.3 × 32.0 mm) (ZRC 2020.0360), Tasek Temengor, south of Banding, Sungai Halong, Perak, colI. Lim KKP and Tan HH, 1–4 November 1993. ***Paratypes***: 1 male (23.9 × 20.0 mm), 3 females (largest 32.8 × 26.9 mm) (ZRC 1995.268), same data as holotype. Others: 1 male (41.2 × 33.8 mm) (ZRC 1995.0269), in logged forest, Hutan Simpanan, Gunung Basor, Sungai Long, off Sungai Pergau, Jeli, Kelantan, 457 m a.s.l., coll. Davison GWH, August 1986.

###### Diagnosis.

Adult carapace width to length ratio 1.20–1.22 (Figs 1G, 2G, 7A, B, 12G); dorsal surface gently convex in frontal view, not inflated (Figs 3G, 7C); frontal margin sinuous (Figs 2G, 7B); suborbital, pterygostomial and sub-branchial regions rugose, pterygostomial region covered with dense setae (Figs 3G, 7C); epigastric cristae distinct, distinctly anterior to sharp postorbital cristae, postorbital cristae with lateral edges low, joining lateral margin through oblique striae (Figs 2G, 7B); external orbital tooth separated from epibranchial tooth by distinct cleft, epibranchial tooth sharp, distinct (Figs 2G, 7B); anterolateral margin distinctly convex (Figs 2G, 7B); posterolateral margin sinuous or with shallow median concavity, distinctly converging towards gently convex, entire posterior carapace margin (Figs 2G, 7B); posterior margin of epistome with triangular median triangle, lateral margin obliquely sloping (Figs 3G, 7C); outer surfaces of third maxillipeds with dense, long stiff setae; ischium subrectangular, with shallow median oblique groove (Figs 3G, 4G, 7C); ambulatory legs not elongate, length to width ratio of merus of fourth ambulatory leg 2.7–2.8 (Figs 1G, 7A, 12G); G1 subterminal segment gradually tapering from broad proximal part to slender distal part, without distinct shelf-like structure along gently concave outer margin; terminal segment gently curved outwards (from median part of sternum), ca. half length of subterminal segment, surfaces with short setae (Fig. 11A–C, E–G); G2 slightly longer than G1, distal segment long, about half length of basal segment (Fig. 11D, H). Female pleon ovate; somites 3–6 progressively narrower; telson semi-circular (Fig. 13G). Vulvae large, on anterior half of sternite 6, adjacent to suture with sternite 5, lateral sternal vulvar cover semi-circular (Fig. 14G).

###### Etymology.

The species is named after the first post-independence director of the National Museum of Singapore (the renamed Raffles Museum) (1967–1973), the late Eric Alfred. A very active freshwater ichthyologist, he collected many of the freshwater crabs in the museum. Eric was a good friend and provided wise counsel and help even years after he stepped down and took over the directorship of the Singapore Maritime Museum.

###### Remarks.

Ng (1995: 250) commented that the G1 terminal segment of the males from Perak “seems to be somewhat longer and more slender” compared to typical *J. tahanensis* but incorrectly attributed it to variation. The differences are actually consistent, and it is here recognised as a separate species, *J. erici* sp. nov. See remarks for *J. booliati* sp. nov. for differences with allied taxa.

###### Distribution.

*Johora erici* sp. nov. is known thus far only from highland streams in northern Perak and Kelantan (Fig. 15).

###### Conservation.

The conservation status for *J. erici* is unclear as we have too few specimens, although it appears to have a relatively wide range in the mountains. It should be best categorised as data deficient for the time being (see Cumberlidge et al. 2009).

##### Johora michaeli sp. nov.

Taxon classificationAnimaliaDecapodaPotamidae

D2FEFB8B-6DD7-5031-9869-2CF249A17299

http://zoobank.org/4CA305BB-71CF-49E5-9559-24BB4019660C

Figures 1H , 2H , 3H , 4H , 5H , 9I–L , 12H , 13H , 14H

###### Material examined.

***Holotype***: male (22.7 × 19.2 mm) (ZRC 2020.0361), waterfalls at rock pools, ca. 5 minutes walk upstream from chalets, Sekayu Waterfall, 4°59'35"N, 102°56'50"E, Terengganu, coll. Tan HH, 21 October 1998. Paratype: 1 female (29.9 × 25.0 mm) (ZRC 2010.0047), same data as holotype. Others: 1 female (30.8 × 25.2 mm) (ZRC 1984.6794), Gunung Padang, Terengganu, 4°50'55.7"N, 102°52'1.9"E, coll. Hislep JSA, 1952. All locations in Peninsular Malaysia.

###### Diagnosis.

Adult carapace width to length ratio 1.20–1.22 (Figs 1H, 2H, 12H); dorsal surface gently convex in frontal view, not inflated (Fig. 3H); frontal margin almost straight (Fig. 2H); suborbital, pterygostomial and sub-branchial regions rugose, pterygostomial region covered with dense setae (Fig. 3H); epigastric cristae distinct, distinctly anterior to sharp postorbital cristae, postorbital cristae with lateral edges low, not joining lateral margin (Fig. 2H); external orbital tooth separated from epibranchial tooth by distinct cleft, epibranchial tooth sharp, distinct (Fig. 2H); anterolateral margin distinctly convex (Fig. 2H); posterolateral margin with median concavity or sinuous, distinctly converging towards gently convex to almost straight, entire posterior carapace margin (Fig. 2H); posterior margin of epistome with triangular median triangle, lateral margin obliquely sloping (Fig. 3H); outer surfaces of third maxillipeds with long stiff setae; ischium subrectangular, with shallow median oblique groove (Figs 3H, 4H); ambulatory legs not elongate, length to width ratio of merus of fourth ambulatory leg 2.7–2.8 (Figs 1H, 12H); G1 subterminal segment gradually tapering from broad proximal part to slender distal part, without distinct shelf-like structure along gently concave outer margin; terminal segment almost straight, slightly curved outwards (from median part of sternum), ca. two-thirds length of subterminal segment, surfaces with scattered short setae (Fig. 9I–K); G2 subequal in length to G1, distal segment long, about one-third length of basal segment (Fig. 9L). Female pleon ovate; somites 3–6 progressively narrower; telson subtriangular (Fig. 13H). Vulvae large, on anterior half of sternite 6, adjacent to suture with sternite 5, lateral sternal vulvar cover subtruncate (Fig. 14H).

###### Etymology.

The species is named after the last director of the Raffles Museum (1946–1967), the late Michael Tweedie, an intrepid collector of interesting animals from Malaysia. The author had the pleasure of knowing him, finally meeting him when the refreshed museum opened as the ZRC in 1988; and even after many years since retiring, he retained his great passion for his crabs, snakes, and fish.

###### Remarks.

The holotype male of *Johora michaeli* sp. nov. is not fully adult and it is clear that it can grow larger, with the adult females reaching 30 mm in carapace width. The G1, however, remains diagnostic, with the terminal segment elongate and almost straight (Fig. 9I–K). While this somewhat resembles that of *J. thoi* which is known from the nearby island of Pulau Redang, that of *J. michaeli* is distinctly less elongate and slender, being only about two-thirds the length of subterminal segment. Specimens of *J. thoi* even smaller than the type of *J. michaeli* remain the same G1 terminal segment shape and proportions of adults (Fig. 9E–G) so the differences observed here are independent of size. The vulva of *J. thoi* is diagnostic, the lateral sternal vulvar cover being triangular in shape (Fig. 14C); it is subtruncate in *J. michaeli* (Fig. 14H). The general shape of the G1 terminal segment somewhat resembles that of *J. tahanensis* s. str. (Fig. 8A–C), it is clearly straighter and slenderer in *J. michaeli* (Fig. 9I–K). It is also unlike that of *J. hoiseni* which has a proportionately and even straighter G1 terminal segment (Fig. 8E–G).

The G1 of *J. michaeli* also resembles that of *J. singaporensis* but the terminal segment in this species is longer (Ng 1987: fig. 8A, B). In any case, the carapace of *J. singaporensis* is quite different from that of *J. michaeli* as it is a member of the *J. johorensis* species group (see remarks for the species group under remarks for the genus).

One large female (ZRC 1984.6794) had been collected from Gunung Padang, which is relatively close to the type locality in Sekayu Falls. Both are part of the same mountain system east of Lake Kenyir in Terengganu.

###### Distribution.

*Johora michaeli* sp. nov. is known so far only from highland streams in central Terengganu (Fig. 15).

###### Conservation.

The conservation status for *J. michaeli* should be regarded as vulnerable as it is known from a relatively well protected area that is not subject to development (see Cumberlidge et al. 2009).

### Key to species of *Johora*

**Table:** 

1	Carapace with anterolateral margins strongly convex, branchial regions appear gently swollen, external orbital tooth very acutely triangular; length of flagellum of third maxilliped exopod subequal to width of merus; G1 very stout, terminal segment cone-shape, tapering to relatively sharp tip (Pahang, Malaysia)	***Johora aipooae* (Ng, 1986a)**
–	Carapace with anterolateral margins gently convex, branchial regions not swollen, external orbital tooth broadly triangular; flagellum on third maxilliped exopod longer than width of merus; G1 slender, variable shapes	**2**
2	Frontal regions narrow, appear compressed, frontal margin slightly below level of external orbital tooth in dorsal view; postorbital cristae sharp, extending to epibranchial tooth as one structure; ambulatory legs especially, very long, length of merus more than 5 times maximum width (highlands, above 750 m a.s.l., Pulau Tioman, Malaysia)	***Johora grallator* Ng, 1988**
–	Frontal region not distinctly narrow, frontal margin level with external orbital tooth; postorbital cristae sharp or low, never extending to epibranchial tooth, if joining always through series of interrupted striae; ambulatory legs not prominently elongate, length of merus less than 4.5 times maximum width	**3**
3	Epigastric cristae just slightly anterior of and almost confluent with or indistinctly separated from postorbital cristae; postorbital cristae distinct not high, usually more prominent along median part of carapace, becoming uneven or breaking up into striae and granules laterally, not clearly reaching cervical groove	**4**
–	Epigastric cristae prominently anterior of and clearly separated from postorbital cristae; postorbital cristae high, sharp along entire length to cervical groove	**12**
4	Dorsal carapace surface usually smooth, striae when present very low, at most with scattered very short setae; G1 terminal segment very slender, hook-shaped, subterminal segment neck-like with stout base; carapace and appendages purplish-red or uniformly orange in life (Pulau Tioman, Malaysia)	**5**
–	Dorsal carapace surface with scattered granules and striae especially along lateral margins, usually with numerous short stiff setae; Gl terminal segment relatively stouter, various shapes, subterminal segment stout; carapace and appendages brown with patches of pale orange in life (Pulau Tioman and rest of Malay Peninsula)	**6**
5	Ambulatory legs relatively shorter, stouter (dactylus of second leg 5.9–7.5 times longer than broad; second ambulatory leg merus 3.5–4.0 times longer than broad); purplish-red in life (lowland species, 100–300 m a.s.l., Pulau Tioman, Malaysia)	***Johora punicea* (Ng, 1985)**
–	Ambulatory legs relatively slenderer (dactylus of second leg 11.4–15.0 times longer than broad; second ambulatory leg merus 4.3–4.4 times longer than broad); purplish-red in life (montane species, ca. 900 m a.s.l., highlands, Pulau Tioman, Malaysia)	***Johora gua* Yeo, 2001**
6	Large species (adult carapace width 30–45 mm); adults with anterolateral margin strongly convex; frontal and anterolateral regions covered with numerous granules and striae (Pulau Tioman, Malaysia)	**7**
–	Small species (adult carapace width 22–23 mm); adults with anterolateral margin gently convex; frontal and anterolateral regions covered with scattered granules and striae (mainland Peninsular Malaysia)	**8**
7	Adult G1 terminal segment prominently hook-shaped, evenly tapering to tip, longer than half length of subterminal segment, surfaces almost smooth or with low flap, indistinct cleft between terminal and subterminal segments (western Pulau Tioman, Malaysia)	***Johora tiomanensis* (Ng & Tan, 1984)**
–	Adult G1 terminal segment slightly sinuous along distal half, shorter than half length of subterminal segment, with distinct flap on distal part of upper margin, distinct broad cleft between terminal and subterminal segments (eastern Pulau Tioman, Malaysia)	***Johora counsilmani* (Ng, 1985)**
8	G1 straight, slender, terminal segment rod-shaped, as long as subterminal segment, medium size species (Singapore)	***Johora singaporensis* Ng, 1986b**
–	G1 bent in varying degrees between terminal and subterminal segment, terminal segment shorter than subterminal segment, tapered, downcurved and hook-shaped, small to large species (Malaysia)	**9**
9	G1 terminal segment gently but distinctly upcurved (Gunong Pulai, Johor, Malaysia)	***Johora johorensis* (Roux, 1936)**
–	G1 terminal segment straight or hook-shaped	**10**
10	G1 terminal segment prominently curved, sickle-shaped, strongly bent, longer than half length of subterminal segment (Central Highlands, Malaysia)	***Johora gapensis* (Bott, 1966)**
–	G1 terminal segment gently curved, hook-shaped, obliquely bent, half or less than half length of subterminal segment	**11**
11	G1 terminal segment curved, about half length of subterminal segment, with broad cleft between terminal and subterminal segments (Gunong Panti and adjacent highlands, Malaysia)	***Johora murphyi* Ng, 1986b**
–	G1 terminal segment slightly curved, tapered, less than half length of subterminal segment, wide ranging subspecies (Selangor, Negeri Sembilan, eastern Pahang, northern Johor, Malaysia)	***Johora intermedia* Ng, 1986b**
12	G1 terminal segment straight to almost straight, long, rod-like, at least two-thirds length of subterminal segment	**13**
–	G1 terminal segment straight to curved and hook-shaped, never as long as subterminal segment	**14**
13	Ambulatory legs relatively longer, length to width ratio of merus of fourth ambulatory leg 3.0–3.2; G1 terminal segment very long, slender, subequal to length of subterminal segment, even in young males (Pulau Redang, Malaysia)	***Johora thoi* Ng, 1990**
–	Ambulatory legs relatively shorter, length to width ratio of merus of fourth ambulatory leg 2.7–2.8; G1 terminal segment almost straight, about two-thirds length of subterminal segment, even in young males (Terengganu, Malaysia)	***Johora michaeli* sp. nov.**
14	G1 terminal segment gently curved outwards	**15**
–	G1 terminal segment straight or nearly so	**17**
15	Adult posterior carapace margin with shallow median indentation; G1 subterminal segment with broad proximal part, tapering relatively suddenly to slender distal part, with low shelf-like structure on outer margin (Bukit Tinggi, Genting Highlands and Fraser’s Hill, Malaysia)	***Johora booliati* sp. nov.**
–	Adult posterior carapace margin gently convex, entire; G1 subterminal segment gradually tapering from broad proximal part to slender distal part, without distinct shelf-like structure along gently concave outer margin (Pahang, Kelantan and Perak, Malaysia)	**16**
16	Frontal margin almost straight; G1 subterminal segment relatively stouter, terminal segment proportionately shorter, stouter (Pahang)	***Johora tahanensis* (Bott, 1966)**
–	Frontal margin sinuous; G1 subterminal segment relatively slenderer, terminal segment proportionately longer, slenderer (Kelantan and Perak, Malaysia)	***Johora erici* sp. nov.**
17	G1 terminal segment with margins subparallel or gently converging for most of length, tip straight to gently curving upwards (Kelantan, Malaysia)	***Johora hoiseni* Ng & Takeda, 1992**
–	G1 terminal segment distinctly tapering, forming conical structure, distal part slightly curved (southern Thailand)	***J. thaiana* Leelawathanagoon, Lheknim & Ng, 2005**

## Supplementary Material

XML Treatment for Johora

XML Treatment for Johora tahanensis

XML Treatment for Johora thoi

XML Treatment for Johora hoiseni

XML Treatment for Johora thaiana

XML Treatment for Johora booliati

XML Treatment for Johora erici

XML Treatment for Johora michaeli
